# Transcriptomic Analysis of Two *Lentinula edodes* Genotypes With Different Cadmium Accumulation Ability

**DOI:** 10.3389/fmicb.2020.558104

**Published:** 2020-09-16

**Authors:** Hailong Yu, Qiaozhen Li, Xiufen Shen, Lujun Zhang, Jianyu Liu, Qi Tan, Yu Li, Beibei Lv, Xiaodong Shang

**Affiliations:** ^1^Institute of Edible Fungi, Shanghai Academy of Agricultural Sciences, National Engineering Research Center of Edible Fungi, Shanghai, China; ^2^Engineering Research Centre of Chinese Ministry of Education for Edible and Medicinal Fungi, Jilin Agricultural University, Changchun, China; ^3^Biotechnology Research Institute, Shanghai Academy of Agricultural Sciences, Shanghai, China

**Keywords:** *Lentinula edodes*, Cd accumulation, RNA-Seq, transporter, MAPK

## Abstract

*Lentinula edodes*, also known as Xiang’gu, is commonly eaten in cultures around the world. However, *L. edodes* is particularly susceptible to enrichment with heavy metals, particularly cadmium (Cd), which is toxic to human health. Understanding the molecular mechanism and mining key genes involved in Cd enrichment will facilitate genetic modification of *L. edodes* strains. Two *L. edodes* genotypes, Le4625 (with higher Cd enrichment capability) and Le4606 (with lower Cd enrichment capability) were used in this study. The Cd concentrations in the mycelia of the tested genotypes differed significantly after Cd (0.1 mg/L) exposure; and the Cd content in Le4625 (1.390 ± 0.098 mg/kg) was approximately three-fold that in Le4606 (0.440 ± 0.038 mg/kg) after 7 h of Cd exposure. A total of 24,592 transcripts were assessed by RNA-Seq to explore variance in Cd accumulation. Firstly, differentially expressed genes (DEGs) were analyzed separately following Cd exposure. In comparison with Ld4625, Ld4606 showed a greater number of Cd-induced changes in transcription. In Ld4606, DEGs following Cd exposure were associated with transmembrane transport, glutathione transfer and cytochrome P450, indicating that these genes could be involved in Cd resistance in *L. edodes*. Next, Le4606 and Le4625 were exposed to Cd, after which DEGs were identified to explore genetic factors affecting Cd accumulation. After Cd exposure, DEGs between Le4606 and Le4625 encoded proteins involved in multiple biological pathways, including transporters on the membrane, cell wall modification, oxidative stress response, translation, degradation, and signaling pathways. Cadmium enrichment in cells may activate MAPK signaling and the anti-oxidative stress response, which can subsequently alter signal transduction and the intracellular oxidation/reduction balance. Furthermore, several possible candidate genes involved in the Cd accumulation were identified, including the major facilitator superfamily genes, heat shock proteins, and laccase 11, a multicopper oxidase. This comparison of the transcriptomes of two *L. edodes* strains with different capacities for Cd accumulation provides valuable insight into the cultivation of mushrooms with less Cd enrichment and also serves as a reference for the construction of engineered strains for environmental pollution control.

## Introduction

*Lentinula edodes*, known as Xiang’gu in China and Shiitake in Japan, is gaining popularity because of its high nutritional and medicinal value. *L. edodes* is rich in essential amino acids, vitamins, and minerals (Chang, 1980), and it contains many pharmaceutical compounds with antibacterial, antioxidant, and immune-enhancing properties (Cao et al., 2013; Dai et al., 2015). *L. edodes* is one of the most economically important cultivated mushrooms, and China is the largest producer and exporter in the world, with an output of 10.43 million tons in 2018 (China Edible Fungi Association [CEFA], 2019).

Heavy metals efficiently accumulate in various edible mushrooms (Liu et al., 2015). Cadmium (Cd) is one of the most toxic heavy metals and may decrease intelligence quotients in children (Khani et al., 2017). Cadmium combines with sulfhydryl groups in proteins and inhibits enzyme activity (Thomet et al., 1999), affecting the cardiovascular system and kidneys (Khani et al., 2017). As Cd can accumulate over the lifetime of an organism (ATSDR, 2007), even a small overdose of Cd can be hazardous to human health. *L. edodes* is readily enriched in Cd (Hatvani and Mécs, 2003), and its high Cd content may adversely affect human health (Mleczek et al., 2017).

Extrinsic and intrinsic factors can affect the accumulation of heavy metals in edible mushrooms. The cultivation medium is the main external factor affecting heavy metal accumulation (Huang et al., 2015; Wang et al., 2016). Mushrooms can produce various organic acids and active enzymes to degrade organic matter, leading to the release of heavy metals, and increasing their absorption. Additionally, trace metal concentrations vary among mushroom species, indicating that genetics are a major intrinsic factor influencing heavy metal accumulation (Huang et al., 2015). Mleczek et al. (2018) analyzed trace elements in 14 mushrooms and found that the contents of toxic deleterious elements varied widely among different mushroom genotypes.

The accumulation of heavy metals (particularly Cd) in fungi has been extensively studied. Some studies report that essential metal ions are replaced by heavy metals through ion exchange (Zadrazil and Brunnert, 1985). In addition, heavy metals are enriched through biosorption processes, such as the complexation of heavy metals with biological macromolecular active groups in cells (Plaza et al., 1996; Marin et al., 1997). For example, metallothionein, which is common in edible mushrooms, may complex with heavy metals, leading to heavy metal accumulation (Qin, 2016). Additionally, active groups on the cell wall, such as sulfhydryl, carboxyl, and hydroxyl groups may react with heavy metal ions to form insoluble substances and deposit heavy metals via physical adsorption or the formation of inorganic precipitates (Xu et al., 2001).

Proteomic and genomic methods have been used to identify key proteins involved in Cd accumulation by *L. edodes* (Zhao et al., 2015; Chen et al., 2016; Dong et al., 2019; Yoo et al., 2019). Although certain oxidases and metallothionein are known to play roles in Cd metabolism, the precise molecular mechanisms underlying Cd accumulation in mushrooms remains unclear. Knowledge of the expression patterns of Cd-related genes in different strains of *L. edodes* following Cd exposure could facilitate the development of improved edible strains with less heavy metal content, as well as strains that readily absorb Cd and could be used for environmental remediation of heavy metal pollution. To investigate Cd response mechanisms in *L. edodes*, two *L. edodes* genotypes with different Cd enrichment capabilities, Le4606 (low enrichment capacity for Cd) and Le4625 (high enrichment capacity for Cd), were cultivated and exposed to Cd, after which RNA-Seq was performed to identify genes involved in Cd transport and metabolism.

## Materials and Methods

### *L. edodes* Mycelium Cultivation and Treatment

*Lentinula edodes* genotypesLe4606 and Le4625 were obtained from the Improved and Standardized Spawn Breeding Center at the Shanghai Academy of Agricultural Sciences (Shanghai, China). Equal doses of both genotypes were inoculated into 100 mL of potato-dextrose broth and cultivated for 7 days with shaking at 120 rpm (25°C). On day 7, CdCl_2_⋅2H_2_O was added to the medium to a final concentration of 0.1 mg/L, as a higher concentration is known to inhibit the growth of mycelium. After Cd exposure for 0, 0.5, and 7 h, mycelial samples were collected and frozen in liquid nitrogen for transcriptome sequencing (three repeats). The mycelium was washed with ddH_2_O and dried at 37°C, after which the dry weight of each sample was determined. The mycelium biomass (four repeats) of Le4606 and Le4625 was 0.23 ± 0.04, 0.37 ± 0.02, 0.39 ± 0.04 g and 0.45 ± 0.02, 0.47 ± 0.19, 0.52 ± 0.04 g after 0, 0.5, and 7 h exposure time. Cadmium content was analyzed by the Shanghai Jiao Tong University Analysis and Testing Center according to the atomic absorption spectrometry method specified in GB5009.15-2014 (National Food Safety Standard-Determination of Cadmium in Food, National Health Commission of the People’s Republic of China).

### Total RNA Isolation, cDNA Library Preparation, and Illumina Sequencing

Total RNA was isolated from each mycelium sample using TRIzol Reagent (Invitrogen, Carlsbad, CA, United States) according to the manufacturer’s instructions. RNA integrity was analyzed by 1.5% denaturing agarose gel electrophoresis, and the RNA concentration and purity were determined with a NanoDrop spectrophotometer (Thermo Fisher Scientific, Waltham, MA, United States). For each sample, 0.75 μg (40 ng/μL) RNA was used for library construction. Libraries were generated using the TruSeq RNA Sample Preparation Kit (Illumina, San Diego, CA, United States). Each mRNA sample was purified and enriched by polyA tail selection and then fragmented. Random hexamer primers were used for first-strand cDNA synthesis, followed by second-strand cDNA synthesis. The cDNA fragments were blunted and ligated to sequencing adaptors. The libraries were then selected according to size (200 bp), and the selected cDNA was amplified via polymerase chain reaction (PCR; 15 cycles) using the Illumina PCR Primer Cocktail. The PCR products were purified (AMPure XP system) and quantified on a Bioanalyzer 2100 system (Agilent Technologies, Santa Clara, CA, United States). The sequencing library was sequenced using a Hiseq Xten system (Illumina). Raw sequencing data were submitted to the National Center for Biotechnology Information (NCBI) Sequencing Read Archive (accession number: SRP256428).

### Data Filtering and Read Mapping

The quality of the raw sequences was evaluated by the FastQC quality control tool^1^. The raw paired-end reads were filtered according to the Phred quality score (Q ≥ 30) and read length (≥50 bp) using SolexaQA software^2^. A reference genome index was established using Bowtie2 software (Langmead et al., 2009), and the clean reads were used for reference (BGI *L. edodes* genomic sequences)-guided transcriptome assembly with Tophat2 software.

### DEG Enrichment Analysis and GO Annotation

The mapped reads were assembled according to the reference genome. Cufflinks software was used to calculate the expression value of each gene, and the gene expression level was standardized by fragments per kb per million mapped reads. DEGSeq was performed to identify the DEGs in Le4606 and Le4625 after Cd stress. The significance and reliability of the multiple-comparison tests were evaluated by calculating the false discovery rate (FDR). The thresholds for significant gene expression differences were FDR ≤ 0.01 and | log2 fold-change| ≥ 1. GO and KEGG function analyses were implemented based on sequence similarities (e-value ≤ 1e^–10^) with proteins in the GO (Gene Ontology)^3^ and KEGG databases (Kyoto Encyclopedia of Genes and Genomes)^4^.

### Validation of Gene Expression by qRT-PCR

To validate the RNA-Seq results, four genes were selected randomly from the significant gene expression patterns and analyzed by quantitative real-time PCR (qRT-PCR). The four selected genes were hypothetical protein (*Le_10013468*), aspartate aminotransferase (*Le_10009629*), amino hydrolase (*Le_10009215*), and hydrophobin 2 (*Le_10001088*). The primer pairs were designed using Primer 3 (V.0.4.0)^5^ as shown in Supplementary Table S1. The actin gene was used as an internal standard.

RNA was isolated as described above. Total RNA (2 μg) was reverse-transcribed using cDNA Synthesis SuperMix (TransGen Biotech, Beijing, China) after genomic DNA was removed (TransScript One-Step gDNA Removal). SYBR green fluorescent dye (TaKaRa, Shiga, Japan) was used to perform the qPCR reactions on an ABI Prism 7500 Real-Time PCR System (Applied Biosystems, Foster City, CA, United States). The PCR conditions were as follows: 95°C for 2 min, 95°C for 15 s (40 cycles), 60°C for 15 s, and 72°C for 30 s. The relative expression value of each gene was calculated using the 2^–ΔΔCt^ method. The experiment was performed using three biological replicates.

### Statistical Analysis

Independent sample *t*-test was performed to analyze the difference between Cd concentrations in two *L. edodes* genotypes. Differences were considered as statistically significant difference when *P* < 0.01. Regression analysis was performed between qPCR and RNA sequencing including all genes for both genotypes at the three time points of Cd treatment. Statistical analysis was performed using SPSS 23.0 (SPSS, Inc., Chicago, IL, United States).

## Results

### Difference in Cd Concentration Between Both Genotypes

**FIGURE 1:**
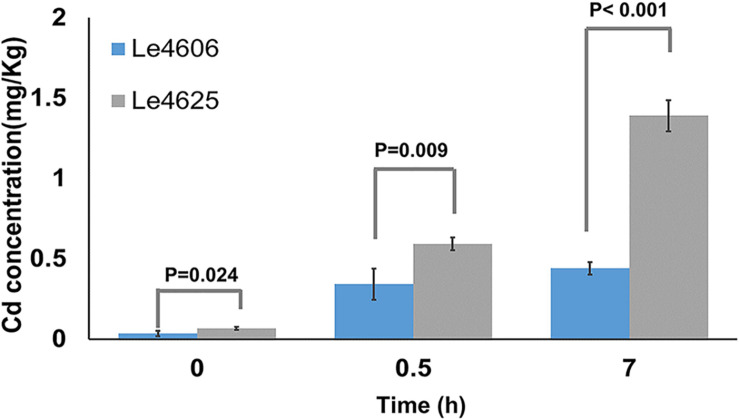
Cd concentrations in *L.edodes* genotypes Le4606 and Le4625 after exposure to Cd (0.1 mg/L) for 0, 0.5, and 7 h.

Mycelia were collected for measurement of Cd content after Cd exposure for 0, 0.5, and 7 h. Initially, the Cd concentrations in Le4606 and Le4625 were 0.035 ± 0.017 and 0.068 ± 0.010 mg/kg, respectively (*P* = 0.024). As shown in Figure 1, a significant difference in the Cd concentration of the selected strains began to appear at 0.5 h, when the Cd concentrations in Le4606 and Le4625 were 0.343 ± 0.097 and 0.593 ± 0.041 mg/kg, respectively (*n* = 4, *P* = 0.009). The difference between the Cd concentrations of the selected strains increased after exposure to Cd for 7 h. The Cd concentration in Le4625 (1.390 ± 0.098 mg/kg) was ∼3-fold that in Le4606 (0.440 ± 0.038 mg/kg; *n* = 4, *P* < 0.001), indicating that Le4625 had a greater capacity for Cd enrichment.

### Transcriptome Sequencing of *L. edodes* and Functional Annotation

RNA-Seq was performed using 18 *L. edodes* cDNA libraries, yielding 797.61 million clean reads. A total of 120.03 Gb of clean data was obtained, and the Q30 value of the base ratio was higher than 90.82% (Supplementary Table S2). The proportion of reads mapping to the *L. edodes* genome was 86.18–90.35% (Supplementary Table S3). The saturation curve of the sequencing data indicated that the sequencing depth was sufficient for transcriptome assembly and expression detection (Supplementary Figure S1). A total of 24,592 transcripts were detected and the genes were annotated via the GO and KEGG pathway databases. The annotated genes were enriched into 14 GO classifications. In the molecular function category, >400 genes were annotated as “binding,” >200 genes were annotated as “catalytic activity,” and >50 genes were annotated as “localization.” Kyoto Encyclopedia of Genes and Genomes pathway analysis showed that the annotated genes were classified into 30 KEGG pathways. Most genes were involved in the “carbohydrate and amino acid metabolism,” “genetic information processing,” and “cellular processes” pathways (Supplementary Figure S2).

### Gene Expression Pattern and RNA-seq Validation by qPCR

**FIGURE 2:**
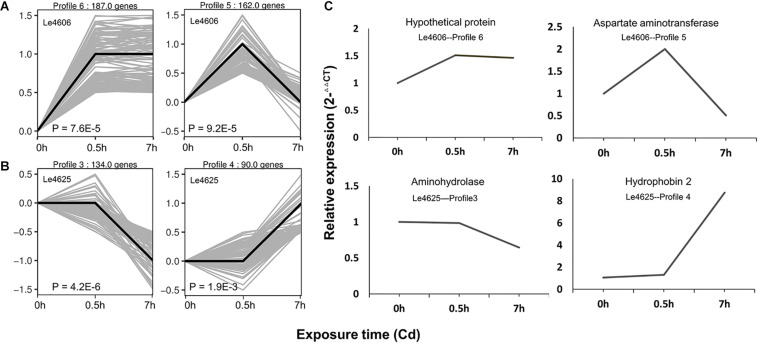
Expression patterns of DEGs and the qRT-PCR validation. **(A)** Significant expression patterns of DEGs in Le4606. **(B)** Significant expression patterns of DEGs in Le4625. **(C)** qRT-PCR validation of DEGs in Le4606 (patterns 5 and 6) and 4605 (patterns 3 and 4).

Based on gene expression patterns, DEGs of Le4606 and Le4625 at different time points were clustered in eight profiles. The profiles displayed considerable difference in the gene expression patterns of the selected genotypes after Cd exposure (Figure 2). In Le4606, 349 DEGs were significantly up-regulated at 0.5 h (Profiles 6 and 5, *P* < 0.001) (Figure 2A). In Le4625, 134 genes were significantly down-regulated and 90 genes were significantly up-regulated at 7 h (Profiles 3 and 4, *P* < 0.001) (Figure 2B). These results suggest a delay in the transcriptional responses of Le4625 to Cd stress, as compared to Le4606.

To validate the expression data obtained by RNA sequencing, one gene from each gene expression pattern was randomly selected for qPCR validation. For each gene, the expression count values from the transcriptome data exhibited an expression profile similar to that shown by the qPCR results (Figure 2C), suggesting that reliable expression results were generated via RNA-seq. The results of this analysis revealed a strong correlation between the RNA-seq and qPCR data (Supplementary Figure S3).

### DEGs in the Same Genotype After Cd Exposure

**FIGURE 3:**
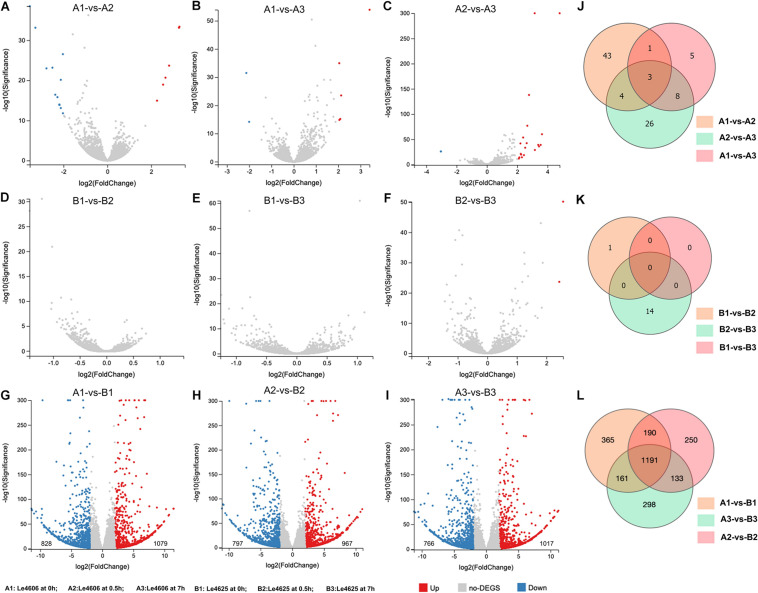
Statistics for differentially expressed genes between Le4606 and Le4625. **(A–C)** Volcano plots of the pairwise comparison of DEGs between three treatments (Cd exposure for 0, 0.5, and 7 h) in the Le4606 genotype. **(D–F)** Volcano plots of the pairwise comparisons of DEGs between three treatments (Cd exposure for 0, 0.5, and 7 h) in the Le4625 genotype. **(G–I)** Volcano plots of DEGs between Le4606 and Le4625 after Cd exposure for 0, 0.5, and 7 h. **(J–L)** Venn analysis of intra-genotype and inter-genotype DEGs for Le4606 and Le4625.

**FIGURE 4:**
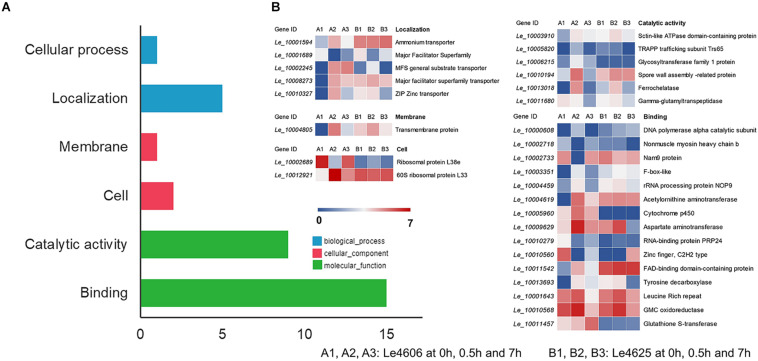
GO classification and transcript abundance of DEGs in Le4606 after Cd exposure. **(A)** GO classifications of DEGs. **(B)** Heatmap of genes in different GO classifications.

To detect genes that may respond to Cd exposure, DEGs before and after Cd exposure were analyzed, with the filtration criteria of ≥2 fold-change and *P* ≤ 0.05. The number of DEGs identified for Le4606 (Figures 3A–C) was greater than that of Le4625 (Figures 3D–F). As shown in Figures 3J,K, comparisons of gene expression at 0, 0.5, and 7 h revealed sets of 51 DEGs (0 h vs 0.5 h) and 17 DEGs (0 h vs 7 h) in Le4606, and four DEGs were detected both at 0.5 and 7 h. These DEGs mainly involved in six GO classifications (Figure 4A), and the transcript abundance of the genes is shown in the heatmap in Figure 4B. *Le_10001689*, and *Le_10002245* were annotated as MFS general substrate transporters with the identity of 72.94 and 92.9%, respectively. *Le_10008273* was annotated as an MFS transporter (identity = 100%) (Table 1). The transcripts of *Le_10008273* and *Le_10002245* were significantly enriched in Le4606 after Cd exposure, but their abundance did not change in response to Cd exposure in Le4625. *Le_10001689* showed similar expression patterns in Le4606 and Le4625; it was abundant before Cd exposure, but its abundance was greatly reduced at 0.5 h and recovered at 7 h. *Le_10004805* was annotated as a transmembrane protein (identity = 99.57%); it was not detected in Le4606 at 0 h, but its expression was significantly increased at 0.5 h and 7 h.

**TABLE 1:** BLASTX results for function description of DEGs involved in different GO classification and KEGG pathways.

**Gene ID**	**Length**	**Accession**	**Description**	**Species**	**E-value**	**Identity (%)**
*Le_10000158*	1744	AGO04566.1	Laccase 11	*Lentinula edodes*	0	96.64
*Le_10000555*	1793	GAW03244.1	Subtilisin-like protein	*Lentinula edodes*	0	99.32
*Le_10000674*	2632	GAW01678.1	Hypothetical protein LENED_003286	*Lentinula edodes*	0	92.86
*Le_10000745*	1027	GAW00691.1	Formate dehydrogenase	*Lentinula edodes*	0	90.70
*Le_10000761*	933	GAW00704.1	Nadp-aldehyde dehydrogenase	*Lentinula edodes*	0	99.66
*Le_10000923*	1262	GAW04578.1	Oxidase-like protein	*Lentinula edodes*	0	89.83
*Le_10000926*	1036	GAW04578.1	Oxidase-like protein	*Lentinula edodes*	9 E-164	71.69
*Le_10001125*	1802	GAW02849.1	Glycoside hydrolase family 13 protein	*Lentinula edodes*	0	99.82
*Le_10001490*	475	GAV99124.1	Small heat shock protein	*Lentinula edodes*	8 E-113	98.06
*Le_10001950*	1839	GAV98680.1	MFS general substrate transporter	*Lentinula edodes*	0	89.98
*Le_10002240*	1518	GAW00057.1	Cytochrome P450	*Lentinula edodes*	0	99.60
*Le_10002241*	600	GAW00058.1	Cytochrome P450	*Lentinula edodes*	1 E-140	97.95
*Le_10002242*	1076	GAW00058.1	Cytochrome P450	*Lentinula edodes*	0	94.61
*Le_10002334*	3071	GAW06274.1	Subtilisin-like protein	*Lentinula edodes*	0	100
*Le_10002462*	1805	GAW07517.1	Non-ribosomal peptide synthetase	*Lentinula edodes*	0	99.78
*Le_10002510*	811	GAW00627.1	Cytochrome P450	*Lentinula edodes*	6 E-172	89.05
*Le_10002534*	1067	GAW00123.	Carbohydrate esterase family 8 protein	*Lentinula edodes*	0	100
*Le_10002545*	945	GAW05932.1	Delta2-dienoyl- -isomerase	*Lentinula edodes*	2 E-169	95.85
*Le_10002801*	1552	KIK50957.1	Hypothetical protein GYMLUDRAFT_234401	*Gymnopus luxurians*	0	85.55
*Le_10003014*	945	GAW00837.1	RTA1 like partial	*Lentinula edodes*	0	99.03
*Le_10003230*	771	GAW09472.1	Winged helix DNA-binding domain-containing partial	*Lentinula edodes*	1 E-134	98.81
*Le_10003290*	1094	GAT45766.1	Cytochrome P450	*Mycena chlorophos*	1 E-98	50
*Le_10003441*	956	BAH57971.1	White collar photoreceptors-like protein	*Lentinula edodes*	0	99.68
*Le_10003459*	1717	GAW08025.1	Aldolase	*Lentinula edodes*	0	77.22
*Le_10003620*	1335	KIK57629.1	Glycoside hydrolase family 7 protein	*Gymnopus luxurians*	0	83.56
*Le_10004084*	1802	GAW09532.1	L-ascorbate oxidase	*Lentinula edodes*	0	99.61
*Le_10004182*	1140	GAW09968.1	CMGC MAPK protein kinase	*Lentinula edodes*	0	100
*Le_10005960*	1024	GAW09426.1	Cytochrome p450	*Lentinula edodes*	0	90.50
*Le_10006253*	829	GAW01409.1	Enoyl- hydratase isomerase	*Lentinula edodes*	0	99.63
*Le_10006364*	1369	GAW03276.1	Sugar transporter	*Lentinula edodes*	0	92.05
*Le_10006934*	1927	GAW08207.1	Siderophore iron	*Lentinula edodes*	0	99.84
*Le_10007179*	1143	GAW05089.1	O-methylsterigmatocystin oxidoreductase	*Lentinula edodes*	0	90.33
*Le_10007538*	1671	GAW10045.1	Cytochrome P450	*Lentinula edodes*	0	75.19
*Le_10007587*	628	KAE9402914.1	Hypothetical protein BT96DRAFT_917863	*Gymnopus androsaceus*	5 E-47	46.23
*Le_10007607*	2348	GAW00202.1	Gata-type sexual development transcription factor	*Lentinula edodes*	0	99.26
*Le_10007614*	2159	GAW00206.1	OPT oligopeptide transporter	*Lentinula edodes*	0	90.04
*Le_10007938*	847	GAW05244.1	Hypothetical protein LENED_007086	*Lentinula edodes*	3 E-94	97.75
*Le_10008178*	1122	GAW06450.1	Carbohydrate esterase family 8 protein	*Lentinula edodes*	0	99.43
*Le_10008419*	1741	GAV98662.1	Winged helix DNA-binding domain-containing partial	*Lentinula edodes*	0	98.70
*Le_10008442*	899	GAV98667.1	Di-copper center-containing protein	*Lentinula edodes*	0	99.66
*Le_10008653*	1839	GAW04922.1	Family S53 protease	*Lentinula edodes*	0	96.50
*Le_10008994*	1064	KIK68569.1	Hypothetical protein GYMLUDRAFT_36029	*Gymnopus luxurians*	0	72.41
*Le_10009137*	1052	GAW03460.1	Glycoside hydrolase family 3 protein	*Lentinula edodes*	0	98.72
*Le_10009468*	2012	GAW04907.1	Autophagy-related protein 11	*Lentinula edodes*	0	96.29
*Le_10009716*	1582	GAW02369.1	Hypothetical protein LENED_004021	*Lentinula edodes*	0	93.05
*Le_10009935*	835	KIK57209.1	Hypothetical protein GYMLUDRAFT_46454	*Gymnopus luxurians*	7 E-146	80.33
*Le_10010320*	731	GAW06466.1	Volvatoxin a2 precursor	*Lentinula edodes*	7 E-136	99.11
*Le_10010322*	777	GAW06464.1	Delta-endotoxin	*Lentinula edodes*	5 E-167	99.21
*Le_10011064*	823	GAW04539.1	Metalloprotease atp23	*Lentinula edodes*	8 E-136	94.40
*Le_10011313*	1454	GAW07580.1	Cytochrome p450	*Lentinula edodes*	0	86.57
*Le_10011530*	1778	GAW04836.1	Pheromone receptor	*Lentinula edodes*	0	89.33
*Le_10012063*	1842	GAW00418.1	Hsp70 family chaperone lhs1	*Lentinula edodes*	0	99.67
*Le_10012169*	1537	GAW03715.1	Hypothetical protein LENED_005459	*Lentinula edodes*	5 E-86	75.36
*Le_10012258*	1540	GAW07216.1	Amid-like NADH oxidoreductase	*Lentinula edodes*	0	89.56
*Le_10012618*	1982	GAW01308.1	Transcriptional activator protein acu-15	*Lentinula edodes*	0	98.05
*Le_10012891*	1509	AGL07749.1	Pheromone receptor	*Lentinula edodes*	0	84.18
*Le_10013475*	1903	GAW03307.1	MFS sugar transporter	*Lentinula edodes*	0	100
*Le_10001594*	1439	GAV99023.1	Ammonium transporter	*Lentinula edodes*	0	90.93
*Le_10001689*	1338	PBK72718.1	MFS general substrate transporter	*Armillaria solidipes*	0	72.94
*Le_10002245*	1494	GAW00062.1	MFS general substrate transporter	*Lentinula edodes*	0	92.90
*Le_10008273*	506	GAW08802.1	MFS transporter	*Lentinula edodes*	4.00 E-95	100
*Le_10003018*	2205	GAW00833.1	hypothetical protein	*Lentinula edodes*	0	98.02
*Le_10008353*	1372	KIK61611.1	hypothetical protein	*Gymnopus luxurians*	3.00 E-67	58.13
*Le_10009735*	704	GAW02386.1	PLC-like phosphodiesterase	*Lentinula edodes*	6.00 E-136	92.61
*Le_10008662*	939	GAW04928.1	aromatic peroxygenase	*Lentinula edodes*	3.00 E-171	90.23
*Le_10010877*	1186	GAW07382.1	hypothetical protein LENED_009369	*Lentinula edodes*	0	94.60
*Le_10003609*	686		general substrate transporter	*Gymnopus luxurians*	2.00 E-99	77.78
*Le_10006404*	1640	GAW08791.1	general substrate transporter	*Lentinula edodes*	0	95.51
*Le_10009268*	1393	GAW03558.1	auxin efflux carrier	*Lentinula edodes*	0	96.08
*Le_10009463*	1073	GAW04909.1	H + Ca2 + exchanger Vxc1-like protein	*Lentinula edodes*	6.00 E-149	98.28
*Le_10014087*	2202	GAW05649.1	DUF803-domain-containing protein	*Lentinula edodes*	0	94.43
*Le_10004339*	3751	GAW06961.1	eukaryotic translation initiation factor 5b	*Lentinula edodes*	0	99.06
*Le_10008377*	2128	GAV98632.1	ribosomal protein L4	*Lentinula edodes*	4.00 E-172	99.63
*Le_10008329*	448	GAV99124.1	small heat shock protein	*Lentinula edodes*	1.00 E-69	66.45
*Le_10008190*	2253	KXN89035.1	Ubiquitin-activating enzyme E1-like protein	*Lentinula edodes*	2.00 E-164	59
*Le_10005860*	548	KIK71029.1	hypothetical protein	*Gymnopus luxurians*	2.00 E-67	74.21
*Le_10013253*	1094	GAW04307.1	antiviral protein	*Lentinula edodes*	0	92.78
*Le_10004805*	735	GAW07683.1	transmembrane protein	*Lentinula edodes*	3.00 E-130	99.57
*Le_10000608*	2253	GAW04482.1	DNA polymerase alpha catalytic subunit	*Lentinula edodes*	0	91.90
*Le_10010279*	2738	GAW06502.1	rna-binding protein prp24	*Lentinula edodes*	0	82.70
*Le_10002733*	1012	GAW00949.1	nam9 protein	*Lentinula edodes*	0	100
*Le_10004459*	2394	GAV99720.1	rRNA processing protein nop9	Lentinula edodes	0	99.80
*Le_10010568*	2446	GAW09218.1	gmc oxidoreductase	*Lentinula edodes*	0	99.86
*Le_10011457*	786	ESK91169.1	glutathione transferase fungal specific class A	*Moniliophthora roreri MCA 2997*	2.00 E-54	43.67

The BLASTX results showed that *Le_10011457* has a thioredoxin-like superfamily domain, and the protein has 43.67% identity with a glutathione transferase (GST) fungal specific class A protein (Table 1). Glutathione transferases are a family of multifunctional enzymes that play important roles in combating abiotic stresses, including heavy metal stress, through amelioration of oxidative damage (Bartling et al., 1993). Before Cd exposure, *Le_10011457* transcript abundance in Le4606 was greater than that in Le4625. After Cd exposure for 7 h, the *Le_10011457* expression level in Le4606 was twice that observed at 0 h. In addition, the expression pattern of cytochrome P450 gene *Le_10005960* was similar to that of *Le_10011457*. The *Le_10005960* transcript abundance in Le4606 was higher than that in Le4625, and *Le_10005960* expression was up-regulated nearly two-fold after Cd exposure for 0.5 h in Le4606. Cytochrome P450 enzymes can detoxify natural and environmental pollutants and allow fungi to grow under a relatively wide range of conditions (Crešnar and Petrič, 2011).

The abundance of *Le_10010568* was down-regulated in Le4606 and Le4625 after 7 h of Cd exposure. *Le_10010568* is a glucose–methanol–choline (GMC) oxidoreductase that is expressed by many organisms and plays an auxiliary roles in lignocellulose degradation in fungi (Sützl et al., 2019). The genes annotated as processing binding functions also participate in DNA synthesis *(Le_10000608*, DNA polymerase alpha catalytic subunit), RNA splicing *(Le_10010279*, RNA-binding protein prp24), and protein translation (*Le_10002733*, Nam9 protein; *Le_10004459*, rRNA processing protein Nop9). In Le4606, *Le_10010279* and *Le_10002733* were significantly down-regulated at 0.5 h, whereas *Le_10000608* and *Le_10004459* expression levels were increased by 100% in comparison with those measured at 0 h. However, Cd exposure did not induce changes in the transcript abundance of *Le_10010279*, *Le_10002733*, *Le_10000608* or *Le_10004459* in Le4625.

### GO and KEGG Analysis of DEGs Between Le4606 and Le4625

Genetics are an important factor affecting Cd accumulation, and differences in the transcription levels of particular genes may result in differences in Cd accumulation in fungi. Therefore, DEGs between Le4606 and Le4625 were analyzed. After Cd exposure for 0, 0.5, and 7 h, Le4625 showed 1079, 967, and 1017 up-regulated genes, respectively, in comparison with Le4606, whereas 828, 797, and 766 down-regulated genes were identified, respectively (Figures 3G–I). A Venn diagram was drawn to illustrate the DEGs between the two genotypes after Cd exposure for different time periods (Figure 3L).

After Cd exposure for 0, 0.5, and 7 h, 365, 250, and 298 DEGs, respectively, were identified between Le4606 and Le4625 (Figure 3L). These sets of DEGs were subjected to GO and KEGG analysis (Supplementary Figure S4). At 0.5 and 7 h, the DEGs were mainly annotated as “binding,” “catalytic activity,” and “localization.” At 0.5 h, the KEGG pathway analysis showed that the genes were involved in “translation,” “cell growth and death,” “folding sorting and degradation,” and “carbohydrate metabolism.” The DEGs were mainly involved in “carbohydrate metabolism,” “cell growth and death,” “folding sorting and degradation,” and “amino metabolism” at 7 h.

**FIGURE 5:**
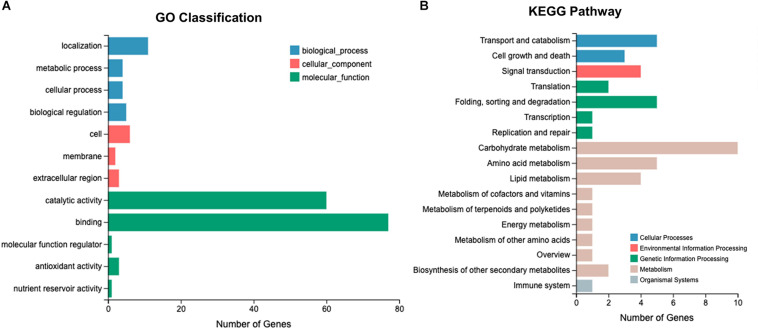
O and KEGG analysis of 1191 DEGs. **(A)** Genes involved in different GO classifications. **(B)** Genes involved in different KEGG pathways.

After Cd exposure for 0, 0.5, and 7 h, 1191 DEGs (fold-change ≥ 2, *P* ≤ 0.05) were identified between Le4625 and Le4606. Among these 1191 DEGs, 680 genes were up-regulated, and 511 genes were down-regulated. The GO classifications of these DEGs revealed their involvement in “metabolic process,” “molecular functions,” “catalytic activity,” “membrane,” and “binding” (Figure 5A). KEGG analysis showed that the DEGs were primarily involved in “carbohydrate metabolism,” “amino acid metabolism,” “lipid metabolism transport and catabolism,” “signal transduction,” and “fold sorting and degradation” (Figure 5B). GO classification and KEGG pathway analysis were performed for the 680 up-regulated genes (Supplementary Figures S5A,B) and 511 down-regulated genes (Supplementary Figures S5C,D) in Le4625. The GO membrane and antioxidant activity functions were specifically enriched in the set of up-regulated genes in Le4625. The KEGG analysis showed that the up- and down-regulated genes were enriched in different metabolic pathways. The up-regulated genes were involved in biosynthesis of other secondary metabolites, as well as metabolism of terpenoids and polyketides.

### Expression of DEGs in Different GO Functions and KEGG Pathways

**FIGURE 6:**
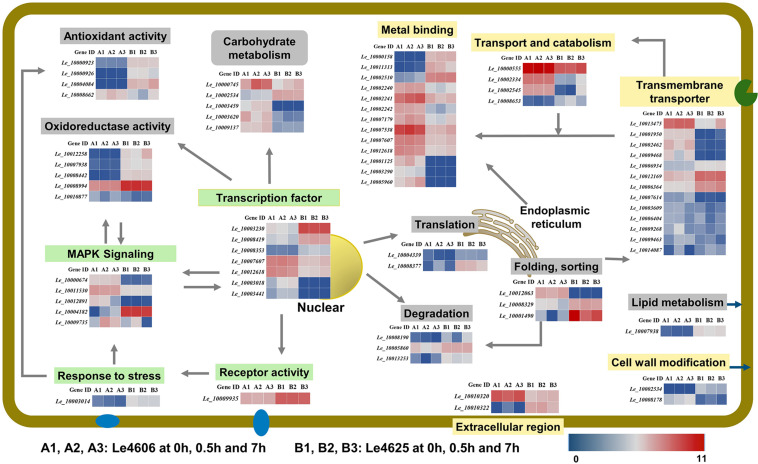
Transcriptional changes of genes involved in Cd enrichment in the two *L. edodes* genotypes. DEGs involved in Cd enrichment are represented in different colors. Genes that may directly respond to heavy metals are shown in a yellow box; Genes involves in signal transduction are shown in a green box. Other genes are shown in gray. For signal transduction, the arrows represent the direction of signaling. For transporters, the arrows represented the directions of Cd transport. For transcriptional factors, the arrows pointed to the products of transcriptions.

To further explore the functions of the DEGs mentioned above, BLASTX analysis was performed based on their sequences (Table 1). Heatmaps of genes involved in GO functions (Supplementary Table S4) and KEGG pathways (Supplementary Table S5) were generated (Figure 6).

#### Expression of Genes Involved in the GO Binding Function

Metal ion binding proteins and transcription factors involved in the GO binding function were selected for analysis. Laccase 11 (*Le_10000158*) gene was involved in copper ion binding, and its transcript was enriched in Le4625. GATA-type sexual development transcription factor (*Le_10007607*), transcriptional activator protein ACU15 (*Le_10012618*), and white-collar-photoreceptor-like protein (*Le_10003441*) were annotated as zinc ion binding genes with transcription factor activity. The expression patterns of *Le_10007607*, *Le_10012618*, and *Le_10003441* were similar, and the transcript abundance of these genes in Le4606 was higher than that in Le4625. Interestingly, *Le_10003441* was expressed in response to Cd stress at 0.5 h in Le4606 but this transcript was not detected in Le4625. In addition, winged-helix DNA-binding domain-containing genes *Le_10003230, Le_10008419, and Le_10008353*, which encode three transcription factors, were relatively enriched in Le4625 at 0 h, 0.5 h and 7 h. A transcript encoding a hypothetical protein annotated as a DNA-binding protein (*Le_10003018*) was enriched in Le4606, but this transcript was not detected in Le4625. *Le_10001125*, which encodes glycoside hydrolase (GH) family 13, an enzyme that bind with cations, was not detected in Le4625 (Figure 6).

#### Expression of Genes Involved in the GO Cellular Component and Localization Function

According to the GO annotation, the volvatoxin a2 precursor (*Le_10010320*) and delta-endotoxin (*Le_10010322*) were identified as being localized in the extracellular region. Both genes showed different expression patterns in Le4606 and Le4625. *Le_10010320* was nearly two-fold more abundant in Le4606 than in Le4625. However, two genes annotated as membrane components, *Le_10003014* (an RTA1-like-gene expressed in response to stress), and *Le_10009935* (a hypothetical protein with receptor activity), were enriched in Le4625 in comparison with Le4606 (Figure 6).

Interestingly, three heat shock protein (HSP)-encoding genes showed different expression levels in Le4606 and Le4625. The transcript abundance of Hsp70 family chaperone *LHS1* (*Le_10012063*) was relatively low in Le4625, but higher in Le4606. Two small HSP genes, *Le_10008329* and *Le_10001490*, were identified. The transcript abundance of *Le_10001490* and *Le_10008329* showed no change in Le4625 after Cd exposure, but they are down-regulated in Le4606 after Cd exposure for 0.5 and 7 h, respectively. Their transcript abundance was 2-fold higher in Le4625 in comparison with Le4606 at 0 h (Figure 6).

#### Expression of Genes Involved in GO Transmembrane Transporter Activity and Cell Wall Modification Functions

Eight genes were annotated as transmembrane transporters. *Le_10012169* (hypothetical protein) and *Le_10006934* (siderophore iron) were enriched in Le4625, whereas the other six genes showed higher transcript abundance in Le4606. In addition, one MFS sugar transporter gene (*Le_10013475*) was up-regulated at 7 h in Le4625. The transcript abundance of another MFS general substrate transporter gene, *Le_10001950* was >6-fold higher in Le4606 in comparison with Le4625. Two carbohydrate esterase family 8 (CE8) genes, *Le_10002534* and *Le_10008178*, may be involved in cell wall modification, but their expression patterns were different. *Le_10008178* was nearly five-fold more enriched in Le4606 in comparison with Le4625, whereas *Le_10002534* showed very low abundance in Le4606, suggesting that these genes have different functions (Figure 6).

#### Expression of Genes Involved in GO Antioxidant and Catalytic Activity Functions

Genes encoding two oxidase-like proteins (*Le_10000923* and *Le_10000926*) and a L-ascorbate oxidase (*Le_10004084*) were annotated as antioxidant genes. All three genes have peroxidase activity and had higher transcript abundance in Le4625; the expression level of *Le_10000923* in Le4625 was three-fold higher than that in Le4606. In contrast, the di-copper-center containing gene *Le_10008442*, amide-like NADH oxidoreductase gene *Le_10012258*, and genes encoding two hypothetical proteins (*Le_10008994* and *Le_10007938*) were annotated as oxidoreductase genes. Interestingly, the peroxidase and oxidoreductase genes were more enriched in Le4625 than in Le4606 at the transcript level. The transcript abundance of *Le_10008662* (aromatic peroxygenase) in Le4606 was two times higher than that in Le4625 at 0.5 h. The transcript abundance of *Le_10010877* (hypothetical protein) in Le4606 was three times higher than that in Le4625 at 7 h (Figure 6).

#### Expression of Genes Involved in the KEGG Metabolism, Translation and Degradation Pathway

Carbohydrate and lipid metabolism pathways were analyzed. Carbohydrate metabolism supplies energy allowing cells to grow or resist stress, and lipid metabolism may affect cell membrane activity. As shown in Figure 6 and Supplementary Table S5, formate dehydrogenase (*Le_10000745*), *CE8* (*Le_10002534*), aldolase (*Le_10003459*), *GH7* (*Le_10003620*), and *GH3 (Le_10009137*) were identified as differentially expressed carbohydrate metabolism genes. With the exception of CE8, the transcripts levels of these genes in Le4606 were greater than those in Le4625. Additionally, in Le4625, the transcript abundance of a hypothetical protein-encoding gene (*Le_10007938*) in a fatty acid elongation pathway involved in cell membrane synthesis was >5-fold higher than that in Le4606 (Figure 6).

Ubiquitin-activating enzyme E1-like protein (*Le_10008190*) and a hypothetical protein gene (*Le_10005860*) were revealed to be involved in ubiquitin mediated proteolysis. *Le_10008190* was down-regulated at 0.5 h (2-fold) in Le4625. *Le_10005860* was down-regulated at 0.5 h in Le4606, and its abundance in Le4625 was not affected by Cd exposure. An antiviral protein (*Le_10013253*) was found to be involved in RNA degradation, and its transcript abundance was lower at 0.5 h in Le4606, but unchanged in Le4625 (Figure 6).

Translation initiation factor 5b (*Le_10004339*) and ribosomal protein L4 (*Le_10008377*), which are both involved in translation, showed higher expression levels in Le4625. *Le_10004339* was down-regulated at 0.5 h both in Le4606 and Le4625, but recovered in Le4625 at 7 h.

#### Expression of Genes Involved in the KEGG Transport and Signaling Pathways

Genes involved in the KEGG signal transduction pathway were annotated as members of the MAPK signaling pathway. Two pheromone receptor genes (*Le_10011530* and *Le_10012891*) and a hypothetical protein-encoding gene (*Le_10000674*) were more highly expressed in Le4606 than in Le4625. The expression levels of both pheromone receptor genes at 0 h were higher than those measured at 7 h in Le4606, and these differences could partially explain the early response of Le4606 to Cd stress. PLC-like phosphodiesterase gene (*Le_10009735*) was up-regulated in Le4606; at 0.5 and 7 h, its transcript abundance was 2 times and 30 times higher, respectively, than that of Le4625 (Figure 6).

Genes involved in transport and catabolism were annotated as members of lysosome- or peroxisome-related biological pathways. The lysosome is an important mediator of metal homeostasis (Blabyhaas and Merchant, 2014). Three lysosome-related genes identified were two subtilisin-like proteins (*Ld_10000555* and *Ld_10002334*) and a family S53 protease (*Ld_10008653*), were identified. The transcript abundance of *Le_10002334* in Le4606 was 3∼5-fold higher than that in Le4625. In contrast, *Le_10002334* expression was enriched in Le4625 after Cd exposure for 7 h. The transcript abundance of *Le_10008653* in Le4625 was 4∼10-fold higher than that in Le4606. The other gene involved in transport and catabolism, *Le_10002545*, was involved in peroxisome. *Le_10002545* expression was enriched in Le4606, and it was up-regulated in Le4625 after Cd exposure for 7 h (Figure 6).

## Discussion

*Lentinula edodes* has high nutritional value and is commonly eaten in many cultures around the world (Cao et al., 2013; Dai et al., 2015). However, *L. edodes*. easily absorbs heavy metals, especially Cd (Hatvani and Mécs, 2003). Cadmium in edible mushrooms can have deleterious effects on human health (Mleczek et al., 2017) and seriously affects the quality of mushrooms (Chiu et al., 1998). Consequently, strict control of Cd content in *L.edodes* is an important consideration in mushroom production. Genetic factors play an important role in Cd enrichment, so determining the molecular mechanism involved in Cd enrichment may facilitate genetic modification of *L. edodes* strains. Therefore, two *L. edodes* genotypes with different capabilities for Cd enrichment, Le4606 and Le4625, were subjected to RNA-Seq analysis. By analyzing the transcriptomes of Le4606 and Le4625, we found candidate genes and pathways that may participate in Cd enrichment.

The Cd enrichment capabilities of Le4606 and Le4625 were analyzed. Firstly, we analyzed the Cd content in Le4606 and Le4625 after Cd exposure for 0, 0.5, 1, 2, 3, 5, 7, and 10 h. Cadmium concentration in mycelium increased rapidly at 0.5 h and it was relative stable in Le4606 after 0.5 h. In addition, the Cd content in Le4625 was highest at 7 h. Therefore, 0.5 and 7 h was apparently important for evaluating the effect of Cd on *L. edodes*. After Cd exposure for 0, 0.5, and 7 h, the Cd concentrations of both strains increased gradually. The Cd content in Le4606 and Le4625 at 0 h was much lower than that at 0.5 and 7 h. A difference in the Cd content of the two genotypes was observed at 0 h. We speculate that the PDB medium may contain a low concentration of Cd, which was absorbed by the mycelium of Le4625. However, the Cd concentration in Le4625 reached 0.593 ± 0.041 mg/kg at 0.5 h, which was ∼9-fold higher than that measured at 0 h, indicating the rapid Cd enrichment capability of the mycelium. The Cd concentration in Le4625 was 1.7-fold or 3.2-fold higher than that in Le4606 after Cd exposure for 0.5 or 7 h, respectively. These results indicate that Le4606 has a low Cd enrichment capacity, whereas Le4625 has a strong ability to absorb and accumulate Cd.

The transcriptomes of both genotypes differed with regard to DEG expression patterns and the number of DEGs. In Le4606, the DEGs were up-regulated at 0.5 h of Cd exposure, whereas changes in DEG expression tended to occur as late as 7 h of Cd exposure in Le4625 (Figure 2). In addition, the number of DEGs in Le4606 was higher than that in Le4625 after Cd exposure for 0.5 and 7 h (Figures 3A–F). These findings suggest that the response of Le4606 to Cd exposure is faster and stronger than that of Le4625. The DEGs induced by Cd exposure in Le4606 were analyzed, and 51 and 17 genes were found to respond to Cd at 0.5 and 7 h, respectively. GO analysis showed that these genes were mainly involved in transporter, binding, and catalytic activity. Three MFS transporters genes (*Le_10001689*, *Le_10008273*, and *Le_10002245*), a transmembrane protein gene (*Le_10004805*), cytochrome and a GST gene (*Le_10011457*) respond to Cd at the early time of 0.5 h. These findings are accordance with a previous study, in which the genes involved in early response to Cd exposure were identified, including MFS transporter, mitogenactivated protein (MAP) kinase, cytochromec peroxidase genes (Zhao et al., 2015). In addition, because genetic factors play an important role in Cd enrichment by *L. edodes*, differences between Le4606 and Le4625 were analyzed on the transcript level. According to the GO and KEGG results, the DEGs participate in many biological pathways, including metal binding, transmembrane transportation, and antioxidant activity. These findings indicate that several biological pathways may play roles in Cd accumulation in *L. edodes*.

The accumulation of heavy metals in edible mushrooms can be summarized as follows: heavy metals are intercepted by the cell wall (Vimala and Das, 2011) and transported via carriers located in the plasma membrane (Severoglu et al., 2013), or they may be chelated by bioactive compounds in the plasma membrane (Bellion et al., 2007). It has been reported that metalloproteins are involved in the uptake, binding, release, and transport of heavy metals *in vivo* (Degtyarenko, 2000). Hence, we selected gene clusters involved in the cell wall, cell membrane, signal transfer, transport, metal ion binding, and other functions based on the GO and KEGG annotations. Next, we analyzed the expression patterns of these gene clusters in the two genotypes using a heatmap. We found that cell wall modification, transmembrane transporter, and metal-binding genes showed different expression patterns in the two genotypes. To understand the biological functions of these DEGs, we conducted a BLASTX search in the NCBI database using their coding DNA sequences (Table 1). The results of this analysis are discussed below.

### Cell Wall Modification Genes May be Involved in Cd Accumulation

Based on reports that heavy metals can be adsorbed onto the cell wall and enriched in fungi (Mo et al., 2010), genes involved in cell wall modification were analyzed. Most carbohydrate esterase family (CE) proteins have been predicted to be involved in the synthesis and remodeling of cell wall components (Lao et al., 2003). A CE8 protein (*Le_10002534*) enriched in Le4625 was homologous to pectin esterase (identity = 68.21%). Pectin methyl esterases (PMEs) belong to the CE8 family and catalyze de-esterification of the homogalacturonan chain of pectin, resulting in de-esterified homogalacturonan. PMEs defend plants from fungal infections, and the tobacco mosaic virus employs PMEs for its systemic spread in plants (Rajulapati et al., 2018). CE8 proteins may respond to enrichment of Cd by remodeling the cell walls of *L. edodes.*

### Genes With Transmembrane Transport Activity

Metal transporters play an important role in metal homeostasis (Bolchi et al., 2011) and can remove toxic metal ions from the cytosol by extrusion or intracellular sequestration (Hall, 2002). Thus, we analyzed genes involved in transmembrane transport, as these protein carriers may participate in transporting Cd (Severoglu et al., 2013). In Le4606, the abundance of four genes annotated as transmembrane transporters was changed after Cd exposure, and three of these genes (*Le_10001689*, *Le_10002245*, and *Le_10008273*) were found to be members of the major facilitator superfamily (MFS). *Le_10002245* and *Le_10008273* were up-regulated in Le4606 after Cd exposure, but not affected in Le4625. In addition, thirteen genes with transmembrane transport activity were differentially expressed between Le4606 and Le4625, and one MFS sugar transporter gene (*Le_10013475*) was gradually enriched in Le4625 by Cd exposure. *Le_10006934* was homologous with a siderophore in *L. edodes* (identity = 99.84%) and the MFS general substrate transporter in *Dendrothele bispora* (identity = 70.02%). The MFS family of transport proteins is essential for the movement of a wide range of substrates, including inorganic anions and cations, across biological membranes (Quistgaard et al., 2016). However, there are distinct families within the MFS, each of which generally transports a single class of compounds. The subfamilies of the MFS genes identified in this study must be determined, and their roles in Cd accumulation will be explored in future studies.

### Genes With Binding Functions

Analysis of the DEGs annotated as binding proteins revealed that most of the identified ion binding genes were zinc binding transcription factors. For example, transcriptional activator protein acu-15 (*Le_10012618*) is located in the nucleus and might function in activating gene expression. The manner in which these transcription factors influence Cd enrichment merits further study. Metallothionein and phytochelatin (cysteine-rich polypeptides with affinity for heavy metals) are involved in regulating metal homeostasis and facilitate cellular detoxification in some edible mushrooms (Haq et al., 2003; Collin et al., 2007). *Le_10000158*, a transcript specifically enriched in Le4625, was found to be homologous to laccase 11 (identity = 96.64%), multicopper oxidase (identity = 96.65%), and ferroxidase (identity = 94.01%) in *L. edodes*. Laccases are Cu-containing glycoproteins that are capable of degrading xenobiotic compounds (Pandey et al., 2018) and are employed by microbes to resist metal ion toxicity by controlling the oxidation states of ions under aerobic conditions (Kaur et al., 2019), but there is no direct evidence support the role it played in Cd response. The role of laccase in Cd metabolism merits further study. In addition, the transcript abundance of several cytochrome P450 enzymes (*Le_10002240*, *Le_10002241*, *Le_10002242*, and *Le_10005960*) was elevated in Le4606 compared with Le4625. Cytochromes P450 monooxygenases are implicated as key enzymes in many fungal processes and mediate detoxification/degradation of xenobiotics (Crešnar and Petrič, 2011). In some studies, a high Cd concentration induced CYP450 in white rot fungus *Phanerochaete chrysosporium* (Zhang et al., 2015) and *Mactra chinensis* (Zhang et al., 2016a). However, the CYP450 superfamily is relatively large, and the subgroups of the CYP450 genes identified in this study and their functions in Cd metabolism merit further study.

### Genes Involved in Protein Translation, Folding, Sorting, and Degradation

In addition to ion binding proteins, genes involved in DNA binding and RNA binding were also identified. After Cd exposure, genes involved in DNA synthesis, RNA splicing, and protein translation were identified in Le4606. *Le_10000608* encodes the DNA polymerase alpha catalytic subunit, which playes an essential role in the initiation of DNA synthesis (Stadlbauer et al., 1994). *Le_10010279* encodes RNA-binding protein PRP24, an essential yeast U6 snRNP protein involved in spliceosome assembly that removes introns from precursor messenger RNA (Kwan and Brow, 2005). *Le_10002733* encodes a Nam9 protein, which is homologous to S4 ribosomal proteins from chloroplasts (Boguta et al., 1992). *Le_10004459* encodes RNA binding protein Nop9, which is required for the production of mature small ribosomal subunit 18S rRNA in yeast (Zhang et al., 2016b). These findings suggest that *L. edodes* responds to Cd exposure at the DNA, RNA and protein levels.

Translation initiation factor 5B (*Le_10004339*) is a universally conserved GTPase that promotes joining of the large and small ribosomal subunits during translation initiation (Murakami et al., 2018). Ribosomal protein L4 (*Le_10008377*) is a typical ribosomal protein that is assembled into the pre-ribosome (Stelter et al., 2015). The abundance of *Le_10004339* and *Le_10008377* in Le4625 was higher than that in Le4606. In addition, in comparison with Le4606, two ubiquitin-mediated proteolysis genes, *Le_10008190* and *Le_10005860*, showed higher transcript abundance in Le4625. Based on these results, the DEGs encoding translation initiation factors and ribosomal proteins play important roles in protein synthesis, and the proteolysis genes are involved in protein degradation. We infer that these DEGs may help maintain the protein balance, or change the protein composition, in mushrooms.

Interestingly, the abundance of two HSP-encoding genes identified in this study differed in Le4606 and Le4625. These HSPs are involved in protein folding, sorting, and degradation to maintain intracellular redox balance under stress. HSPs play important roles in stress responses (Mandelli et al., 2017) and have been found to respond to Cd stress in *Pleurotus eryngii* (Li et al., 2018). Hsp70 family chaperone *LHS1* (*Le_10012063*) was enriched in Le4606 and almost undetectable in Le4625. LHS1 is a chaperon of endoplasmic reticulum in yeast and is involved in protein folding, its expression was higher in the Cd hypersensitive strain (Gardarin et al., 2010). We infer that the Le4606 was sensitive to the increasement of Cd concentration, and *LHS1* was involved in Cd stress and might protect the cells by protein folding. In addition, two small HSPs *Le_10008329* and *Le_10001490*, were identified. The transcript abundance of *Le_10008329* and *Le_10001490* was relatively stable in Le4625. In Le4606, *Le_10001490* was down-regulated at 0.5 h and *Le_10008329* was down-regulated at 7 h. The changes in gene expression suggested a functional diversity between the sHSPs in Cd enrichment. SHSPs are widely present in all kingdoms of life, and they promote protein homeostasis and proteome stability under stressful conditions (Carra et al., 2017). Martín-Folgar and Martínez-Guitarte (2017) analyzed the expression profile of *sHSPs* under Cd exposure in *C. riparius larvae*. Cadmium exposure will change the expression of *sHSPs*, some *sHSPs* genes are rapidly activated by Cd, and some *sHSPs* are induced under long-term cadmium stress. In this study, *HSP27* was downregulated at early time of Cd exposure, but significant upregulated after 24 h of Cd treatment. The authors proved the function for *HSP27* during the long-term response after Cd exposure. The similar expression of *Le_10001490* may indicate its function in the long-term of Cd exposure. In addition, the expression pattern of *Le_10008329* may suggest a relevant function in the early response to Cd enrichment.

### MAPK Signaling Pathway

Plants respond to heavy metal stress by inducing several distinct MAPK pathways (Jonak et al., 2004) that enhance tolerance to Cd stress. Cadmium may activate MPK3 and MPK6 via the accumulation of reactive oxygen species in *Arabidopsis* (Liu et al., 2010). In a previous study, a MAP kinase was found to be involved in responses to 24 h of Cd exposure in *L. edodes* (Zhao et al., 2015). In this study, we identified changes in the expression levels of genes participating in the MAPK signaling pathway following Cd exposure. *Le_10004182* was annotated as a CMGC MAPK protein kinase, a superfamily that is mainly composed of the catalytic domains of serine/threonine-specific and tyrosine-specific protein kinases. The potential role of *Le_10004182* in Cd enrichment will be verified in future studies.

### Anti-Oxidative Stress Response

Peroxidation can facilitate Cd removal via coagulation under alkaline conditions (Wang et al., 2016). Several studies have reported that the antioxidant system contributes to microbial resistance to heavy metals (Guo et al., 2016), thereby allowing fungi to counteract reactive oxygen species accumulation initiated by metals (Zhao et al., 2015). Genes annotated as antioxidants had different expression patterns in the two genotypes investigated in this study. Genes involved in oxidoreductase activity showed greater abundance in Le4625 in comparison with Le4606, with the exception of *Le_10008994*. Additionally, the redox reaction can change intracellular pH and facilitate a reduction in the intracellular Cd concentration. This finding indicates that a high Cd concentration is a stress factor for mushrooms that activates antioxidant mechanisms.

Glutathione transferases have been reported in all organisms, including fungi, and are notable for their roles in protecting cells from oxidative damage. Moreover, GSTs play important roles in abiotic stress responses and heavy metal detoxification reactions (Kumar and Trivedi, 2018). In a previously study, overexpression of a *GST* gene in tobacco resulted in plants that were tolerant to Cd and showed reduces Cd accumulation in the plant biomass (Dixit et al., 2011). In this study, a *GST* gene (*Le_10011457*) was up-regulated after Cd exposure. The role of *Le_10011457* in Cd enrichment in *L. edodes* will be verified in future studies.

In conclusion, *L. edodes* genotypes (Le4606 and Le4625) showed different Cd accumulation capacities, which were attributed to genetic differences. The DEGs between these two genotypes were found to be involved in a variety of biological processes. MFS transporters (*Le_10006934*, *Le_10013475*, *Le_10001689*, *Le_10002245*, and *Le_10008273*), CYP450 enzymes (*Le_10002240*, *Le_10002241*, *Le_10002242*, and *Le_10005960*), and a GST (*Le_10011457*) may be involved in Cd accumulation in mushrooms. Additionally, Cd enrichment in cells may activate MAPK signaling and the anti-oxidative stress response, which play roles in signal transduction and maintaining intracellular redox balance. Further work should be conducted to delineate the functions of the identified genes and elucidate their regulation mechanisms in Cd enrichment. The candidate genes may be applied in gene engineer to alter the Cd enrichment ability of *L. edodes*, to develop edible strains with less Cd.

## Data Availability Statement

The datasets presented in this study can be found in online repositories. The names of the repository/repositories and accession number(s) can be found below: https://www.ncbi.nlm.nih.gov/, SRP256428.

## Author Contributions

BL and XDS conceived the study design. QL was responsible for the sample preparation and RT-PCR. HY, LZ, and JL performed the bioinformatic analysis. HY completed the initial manuscript. QT, YL, and XFS revised the manuscript. All the authors read and approved the final version of the manuscript.

## Conflict of Interest

The authors declare that the research was conducted in the absence of any commercial or financial relationships that could be construed as a potential conflict of interest.

## Footnotes

1 http://www.bioinformatics.babraham.ac.uk/projects/fastqc/

2 http://solexaqa.sourceforge.net/

3 http://geneontology.org/

4 http://www.genome.jp/kegg

5 http://bioinfo.ut.ee/primer3-0.4.0/
