# Supplementary material for: Transcriptomic Analysis of Two Lentinula edodes Genotypes With Different Cadmium Accumulation Ability
Source: Front Microbiol. 2020 Sep 16;11:558104. doi: 10.3389/fmicb.2020.558104 (PMC7526509; doi:10.3389/fmicb.2020.558104)

**Supplementary Figures**

**Supplementary figure 1** The sequencing saturation curve.

Three biological repeats were taken in each treatment for the sequencing. A1-1, A1-2, A1-3 means the 3 repeats;

A1: Le4606 at 0 h; A2: Le4606 at 0.5 h; A3: Le4606 at 7 h; B1: Le4625 at 0 h; B2: Le4625 at 0.5 h; B3: Le4625 at 7 h


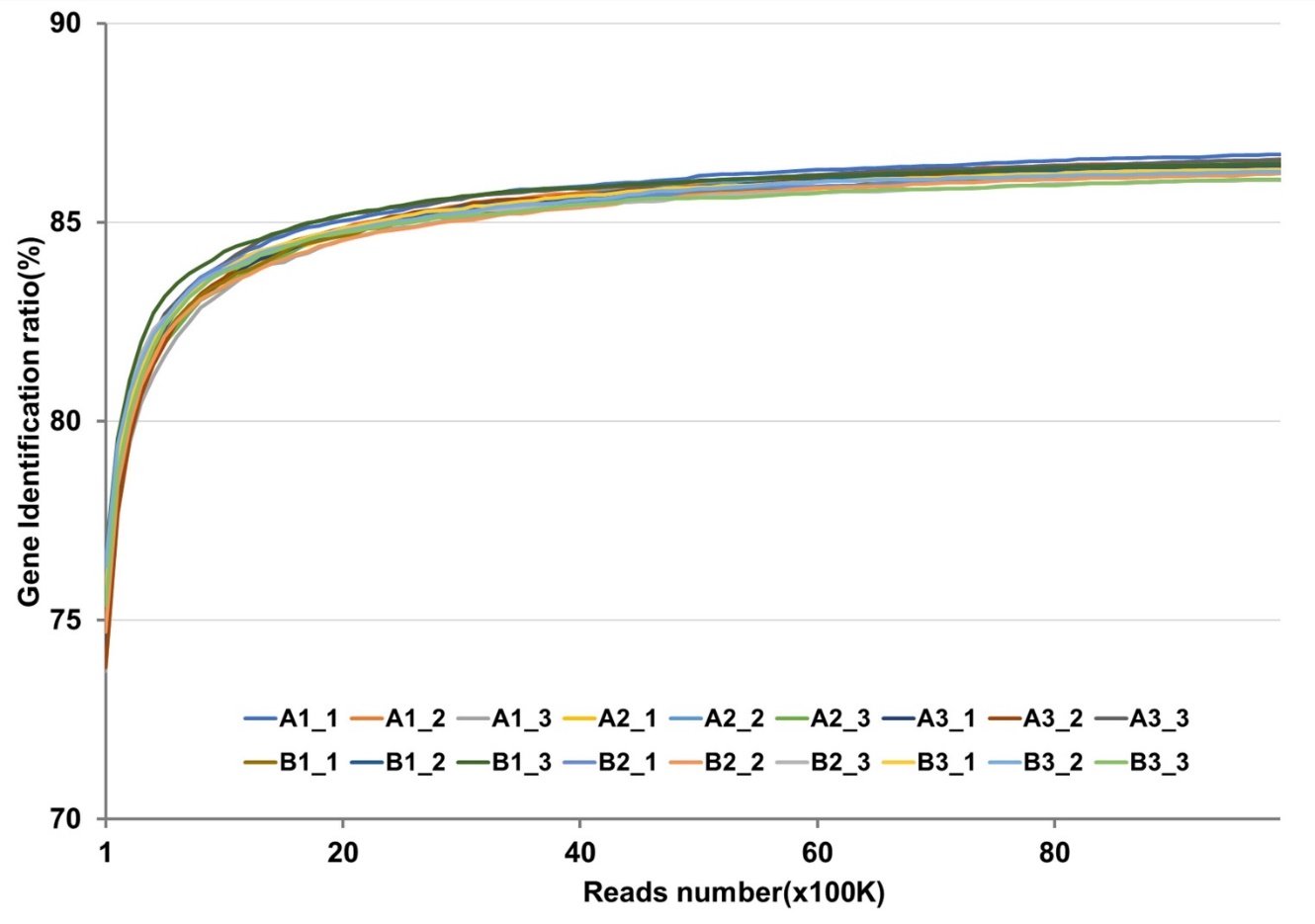


**Supplementary figure 2** The GO classification and KEGG Pathway of the annotated genes.


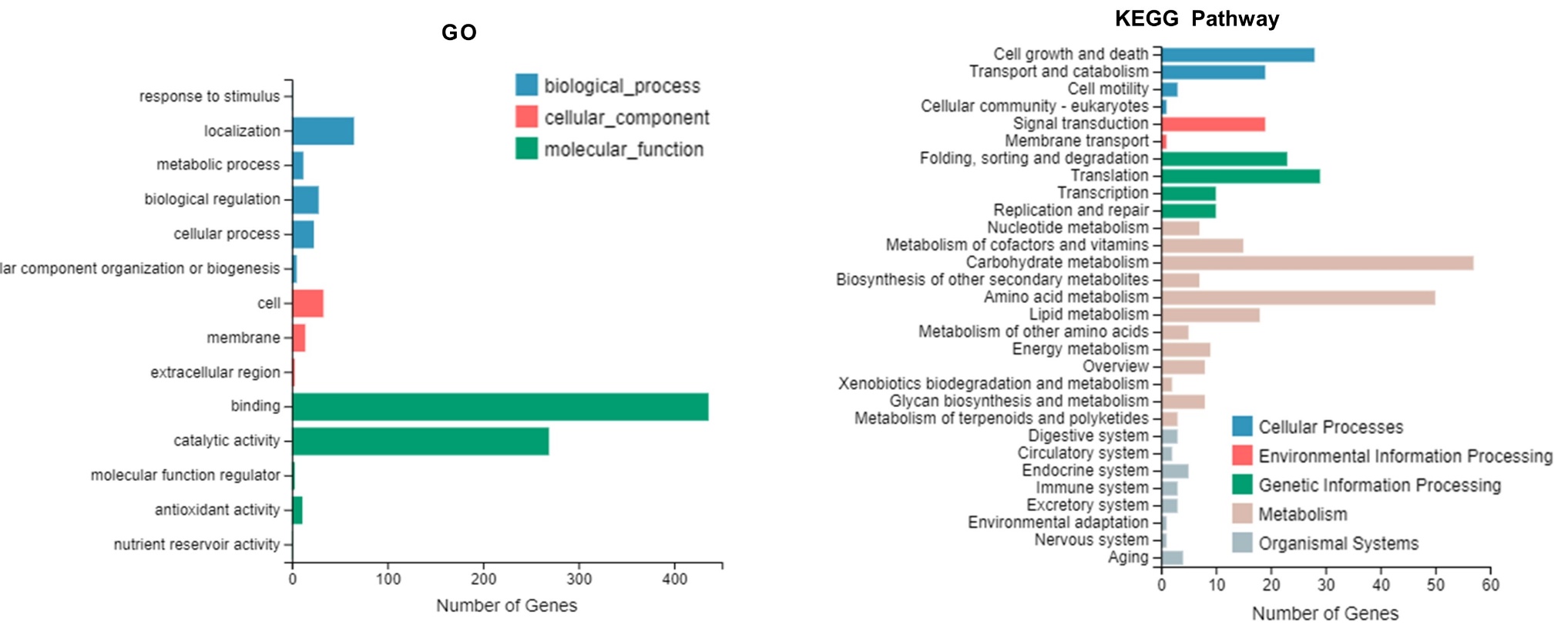


**Supplementary figure 3** The Pearson correlation between the data of RNA sequencing and qPCR.


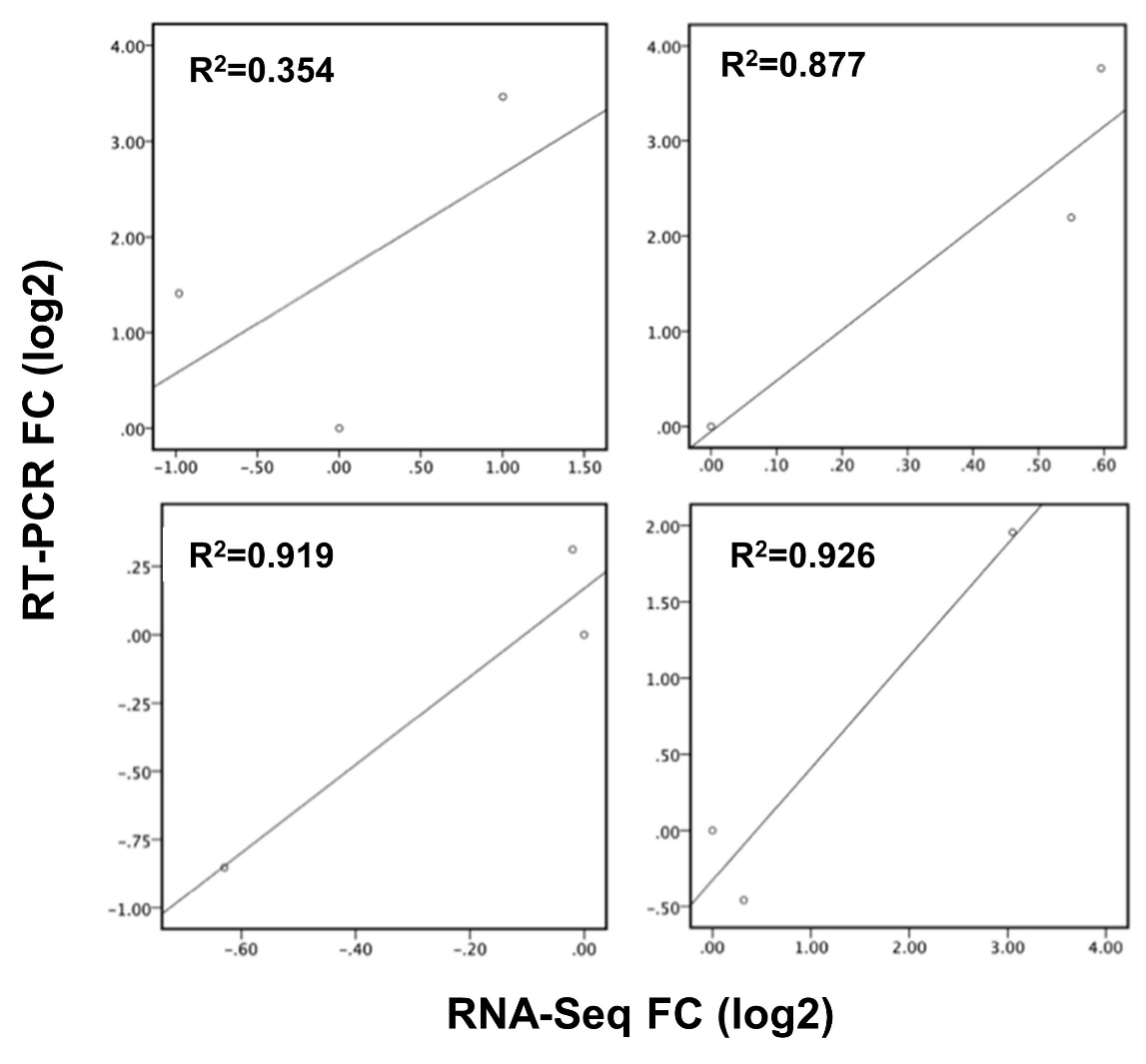


**Supplementary figure 4** GO and KEGG analysis of DEGs between Le4606 and Le4625 after Cd exposure for 0.5 h and 7 h

(A-B) GO annotation and KEGG pathway analysis of the DEGs (250 genes), detected only at 0.5 h between Le4606 and Le4625;

(C-D) GO annotation and KEGG pathway analysis of the DEGs (298 genes), detected only at 7 h between Le4606 and Le4625;

(E-F) GO annotation and KEGG pathway analysis of the DEGs (133 genes), detected only at 0.5 h and 7 h between Le4606 and Le4625.


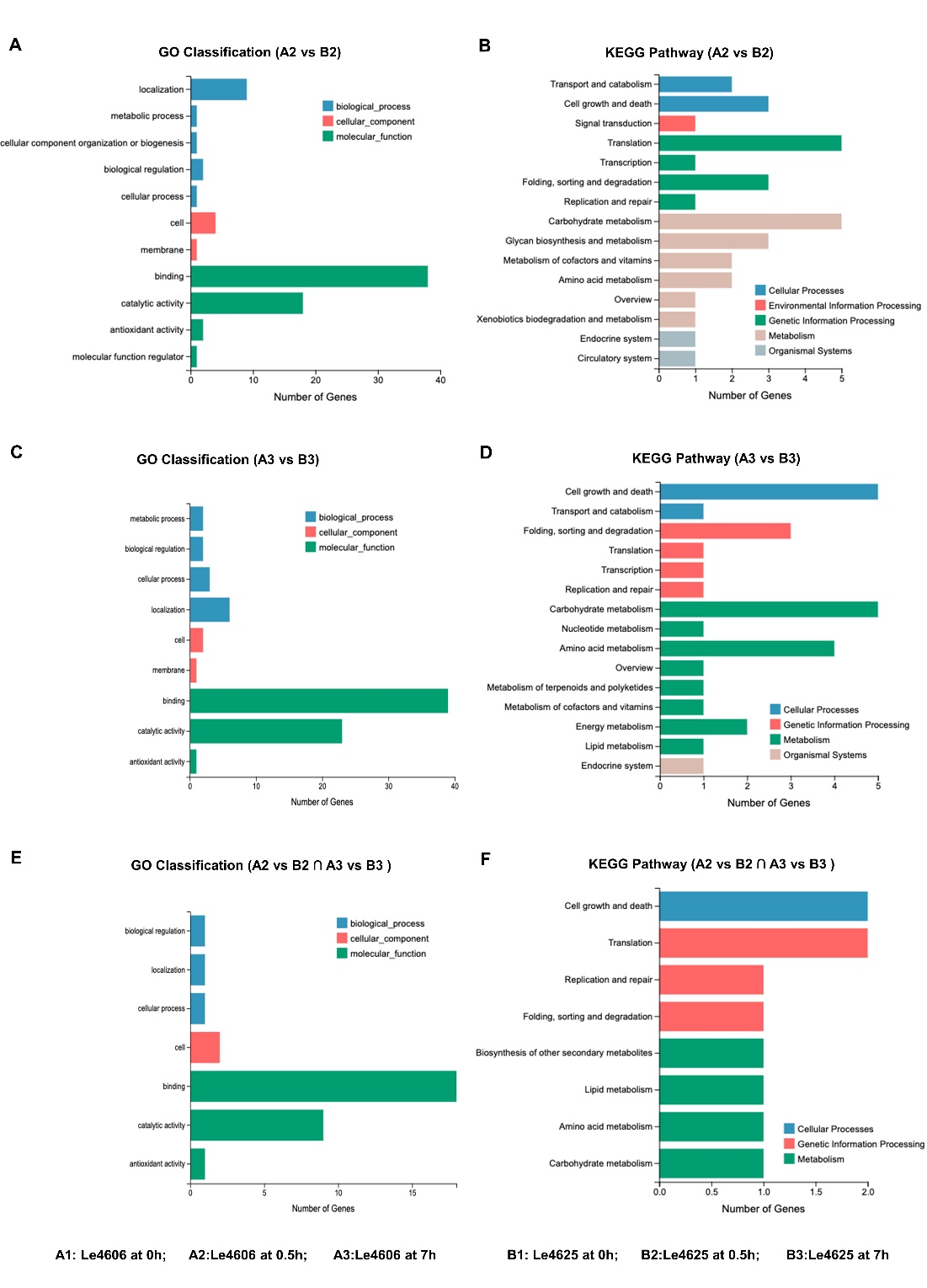


**Supplementary figure 5** The GO classification and KEGG pathway analysis for the up-regulated 680 genes (A and B) and down-regulated 511 genes (C and D).


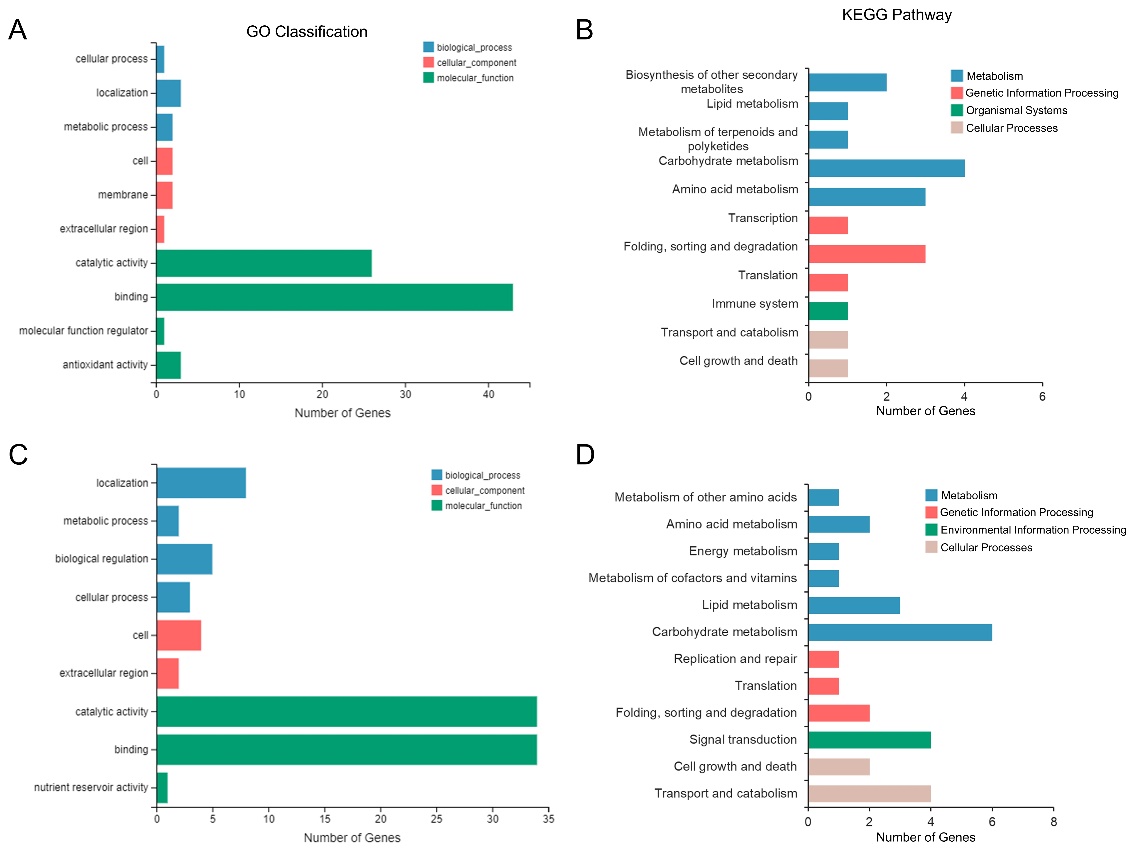

Supplement: Supplementary file 1 [file Data_Sheet_1.DOCX]
