# Supplementary material for: Transcriptomic Analysis of Two Lentinula edodes Genotypes With Different Cadmium Accumulation Ability
Source: Front Microbiol. 2020 Sep 16;11:558104. doi: 10.3389/fmicb.2020.558104 (PMC7526509; doi:10.3389/fmicb.2020.558104)
Supplement: Supplementary file 2 [file Data_Sheet_2.docx]

**Supplementary Tables**

**Supplementary Table 1** Primers for qRT-PCR of the DEGs

| Gene Name | Gene ID | Primer-F | Primer-R |
| --- | --- | --- | --- |
| Aspartate Aminotransferase | *Le_10009629* | GCGACGACAATGATAAGC | AGACCAGTGATAGGCAGAT |
| Hypothetical protein | *Le_10013468* | CGACGACATTAGGAGAATCT | AGACCAGTGATAGGCAGAT |
| Aminohydrolase | *Le_10009215* | AACACTGACACTGAGCATT | TTGACAAGAGCAAGTAGCA |
| Hydrophobin 2 | *Le_10001088* | TATCGCAGCACTGGACAG | AGCAAGTAATCGTCGTTCAA |
| Actin |  | GGAGAAGATTTGGCATCACACA | GAAGAGCGAAACCCTCGTAGA |

**Supplementary Table 2** Quality Statistics of filtered reads

Three biological repeats were taken in each treatment.

| **Sample** | **Total Raw Reads (M)** | **Total Clean Reads (M)** | **Total Clean Bases (Gb)** | **Clean Reads Q20 (%)** | **Clean Reads Q30 (%)** |
| --- | --- | --- | --- | --- | --- |
| **4606-0h-1** | 44.05 | 44.05 | 6.63 | 96.26 | 91.29 |
| **4606-0h-2** | 43.97 | 43.97 | 6.62 | 96.08 | 90.92 |
| **4606-0h-3** | 41.35 | 41.35 | 6.22 | 96.14 | 90.99 |
| **4606-0.5h-1** | 42.12 | 42.12 | 6.34 | 96.25 | 91.2 |
| **4606-0.5h-2** | 47.28 | 47.28 | 7.1 | 96.5 | 91.71 |
| **4606-0.5h-3** | 42.1 | 42.1 | 6.34 | 96.13 | 90.97 |
| **4606-7h-1** | 41.9 | 41.9 | 6.3 | 96.06 | 90.82 |
| **4606-7h-2** | 39.63 | 39.63 | 5.96 | 96.16 | 91.05 |
| **4606-7h-3** | 44.7 | 44.7 | 6.73 | 96.13 | 91.0 |
| **4625-0h-1** | 40.33 | 40.33 | 6.07 | 96.09 | 90.9 |
| **4625-0h-2** | 45.62 | 45.62 | 6.87 | 96.54 | 91.84 |
| **4625-0h-3** | 52.94 | 52.94 | 7.97 | 96.92 | 92.6 |
| **4625-0.5h-1** | 49.92 | 49.91 | 7.51 | 96.9 | 92.57 |
| **4625-0.5h-2** | 40.34 | 40.34 | 6.07 | 96.81 | 92.38 |
| **4625-0.5h-3** | 43.67 | 43.66 | 6.57 | 97.09 | 92.95 |
| **4625-7h-1** | 44.56 | 44.56 | 6.71 | 97.03 | 92.84 |
| **4625-7h-2** | 49.51 | 49.51 | 7.45 | 97.28 | 93.35 |
| **4625-7h-3** | 43.64 | 43.64 | 6.57 | 97.26 | 93.31 |
| **Total** | 797.63 | 797.61 | 120.03 |  |  |

**Supplementary Table 3** Statistics on mapping of the clean reads

Three biological repeats were taken in each treatment.

| **Sample** | **Total Clean Reads** | **Total Mapping (%)** | **Uniquely Mapping (%)** |
| --- | --- | --- | --- |
| **4606-0h-1** | 40495996 | 90.22 | 88.26 |
| **4606-0h-2** | 40133320 | 90.2 | 88.22 |
| **4606-0h-3** | 37618644 | 90.21 | 88.11 |
| **4606-0.5h-1** | 38446476 | 90.1 | 88.01 |
| **4606-0.5h-2** | 43528150 | 89.84 | 87.69 |
| **4606-0.5h-3** | 38289894 | 90.14 | 88.03 |
| **4606-7h-1** | 38002160 | 89.83 | 87.77 |
| **4606-7h-2** | 36071198 | 89.86 | 87.72 |
| **4606-7h-3** | 40857366 | 90.35 | 88.19 |
| **4625-0h-1** | 36705782 | 86.89 | 85.13 |
| **4625-0h-2** | 42262972 | 86.18 | 84.52 |
| **4625-0h-3** | 49568612 | 86.65 | 84.91 |
| **4625-0.5h-1** | 46740700 | 86.71 | 84.94 |
| **4625-0.5h-2** | 37660930 | 86.41 | 84.53 |
| **4625-0.5h-3** | 41084088 | 86.23 | 84.52 |
| **4625-7h-1** | 41860420 | 86.64 | 84.92 |
| **4625-7h-2** | 47124066 | 86.62 | 84.98 |
| **4625-7h-3** | 41572454 | 86.5 | 84.82 |

**Supplementary Table 4** The expression abundance of different expression genes in the KEGG pathways

| Gene ID (gene_Lentinula_  edodes_GLEAN_X) | Pathway Name | FPKM (Log_2_ (X+1)) | | | | | |
| --- | --- | --- | --- | --- | --- | --- | --- |
|  |  | 4606-0h | 4606-0.5h | 4606-7h | 4625-0h | 4625-0.5h | 4625-7h |
| Carbohydrate metabolism | | | | | | | |
| *Le_*10000745 | ko01200//Carbon metabolism; ko00680//Methane metabolism; ko00630//Glyoxylate and dicarboxylate metabolism; | 4.5352 | 7.2076 | 6.5849 | 2.5769 | 3.4078 | 4.0747 |
| *Le_*10002534 | ko00040//Pentose and glucuronate interconversions; | 1.8979 | 2.1784 | 2.2520 | 4.6167 | 4.4460 | 4.2482 |
| *Le_*10003459 | ko01230//Biosynthesis of amino acids; ko01200//Carbon metabolism; ko00030//Pentose phosphate pathway; ko00010//Glycolysis / Gluconeogenesis; ko00051//Fructose and mannose metabolism; ko00680//Methane metabolism;ko00710//Carbon fixation in photosynthetic organisms; | 2.4577 | 3.2403 | 3.2570 | 0.0000 | 0.0000 | 0.0000 |
| *Le_*10003620 | ko00500//Starch and sucrose metabolism; | 4.6377 | 2.8600 | 3.8080 | 1.4222 | 0.7991 | 0.8979 |
| *Le_*10009137 | ko00520//Amino sugar and nucleotide sugar metabolism; | 3.1705 | 3.2464 | 3.8911 | 1.3353 | 1.4751 | 1.4507 |
| Transport and catabolism | | | | | | | |
| *Le_*10000555 | ko04142//Lysosome; | 10.5293 | 10.6672 | 10.3336 | 7.3199 | 7.1310 | 7.8951 |
| *Le_*10002334 | ko04142//Lysosome; | 6.4068 | 6.3044 | 6.2351 | 1.5753 | 1.4507 | 2.4785 |
| *Le_*10002545 | ko04146//Peroxisome; | 4.7996 | 4.8128 | 4.8246 | 0.2829 | 0.0000 | 2.6652 |
| *Le_*10008653 | ko04142//Lysosome; | 0.4647 | 0.2510 | 0.4889 | 1.8732 | 2.3103 | 1.6229 |
| Signal transduction | | | | | | | |
| *Le_*10000674 | ko04013//MAPK signaling pathway; ko03008//Ribosome biogenesis in eukaryotes;ko03013//RNA transport; | 3.0647 | 3.0715 | 3.3472 | 0.4542 | 0.3859 | 0.5721 |
| *Le_*10007587 | ko04392//Hippo signaling pathway - multiple species; ko04391//Hippo signaling pathway -fly; ko04011//MAPK signaling pathway - yeast; ko04390//Hippo signaling pathway; | 4.3655 | 4.7551 | 5.1289 | 1.6584 | 2.1932 | 2.5177 |
| *Le_*10011530 | ko04011//MAPK signaling pathway - yeast; | 5.0118 | 4.7268 | 4.6895 | 2.7232 | 2.6237 | 2.6369 |
| *Le_*10012891 | ko04011//MAPK signaling pathway - yeast; | 3.3195 | 2.9879 | 1.4594 | 0.0000 | 0.0000 | 0.1287 |
| FoLeing, sorting and degradation | | | | | | | |
| *Le_*10001490 | ko04141//Protein processing in endoplasmic reticulum; | 3.5632 | 1.2408 | 3.2006 | 5.8730 | 5.1775 | 5.3870 |
| *Le_*10012063 | ko04141//Protein processing in endoplasmic reticulum; | 4.4044 | 4.1249 | 4.7060 | 0.0000 | 0.0000 | 0.0000 |
| *Le_*10008329 | ko04141//Protein processing in endoplasmic reticulum; | 2.6151 | 2.7463 | 1.8849 | 4.4443 | 4.8391 | 4.5856 |
| *Le_*10008190 | ko04120//Ubiquitin mediated proteolysis; | 0.5227 | 0.2388 | 0.0000 | 1.8653 | 0.6167 | 2.3065 |
| *Le_*10005860 | ko04120//Ubiquitin mediated proteolysis; ko04113//Meiosis - yeast; ko04111//Cell cycle - yeast; ko04914//Progesterone-mediated oocyte maturation; ko04110//Cell cycle; ko04114//Oocyte meiosis; | 3.5282 | 2.4186 | 3.0421 | 4.3448 | 4.3650 | 4.3778 |
| *Le_*10013253 | ko03018//RNA degradation; | 1.0096 | 0.0000 | 0.9758 | 2.6237 | 2.2550 | 2.4033 |
| Lipid metabolism | | | | | | | |
| *Le_*10007938 | ko00062//Fatty acid elongation; ko01040//Biosynthesis of unsaturated fatty acids; ko01212//Fatty acid metabolism; | 0.4612 | 0.2750 | 0.2987 | 2.7662 | 2.5025 | 2.7254 |
| Translation |  |  |  |  |  |  |  |
| *Le_*10004339 | ko03013//RNA transport; | 0.6198 | 0.1678 | 0.0704 | 1.3467 | 1.3353 | 1.2245 |
| *Le_*10008377 | ko03010//Ribosome; | 0.2987 | 1.5705 | 0.4222 | 4.0024 | 3.8646 | 3.1133 |

**Supplementary Table 5** The expression abundance of DEGs in different GO functions

| Gene ID (ene_Lentinula_  edodes_GLEAN_X) | GO Term | FPKM (Log_2_ (X+1)) | | | | | |
| --- | --- | --- | --- | --- | --- | --- | --- |
|  |  | 4606-0h | 4606-0.5h | 4606-7h | 4625-0h | 4625-0.5h | 4625-7h |
| Binding | | | | | | | |
| *Le_*10000158 | [F]GO:0005507//copper ion binding | 0.0612 | 0.0000 | 0.0000 | 3.6997 | 4.0444 | 4.1160 |
| *Le_*10001125 | GO:0043169//cation binding | 3.7520 | 4.5229 | 2.9696 | 0.0000 | 0.0000 | 0.0000 |
| *Le_*10002240 | iron ion binding | 4.3840 | 4.9008 | 4.7738 | 2.3505 | 2.0909 | 2.5599 |
| *Le_*10002241 | GO:0005506//iron ion binding | 6.3074 | 7.1121 | 7.5190 | 4.4096 | 3.5390 | 4.0347 |
| *Le_*10002242 | GO:0005506//iron ion binding | 4.6686 | 4.5439 | 4.2245 | 1.8953 | 1.4418 | 2.1021 |
| *Le_*10002510 | GO:0005506//iron ion binding | 1.1419 | 1.2810 | 1.7341 | 5.6273 | 5.8511 | 5.9013 |
| *Le_*10002801 | GO:0005506//iron ion binding | 0.4365 | 0.2347 | 0.4820 | 1.4005 | 1.3239 | 1.5311 |
| *Le_*10003290 | GO:0005506//iron ion binding | 2.6886 | 2.3822 | 2.1689 | 0.0000 | 0.0000 | 0.0000 |
| *Le_*10005960 | GO:0005506//iron ion binding | 2.9585 | 3.7122 | 4.8895 | 0.0000 | 0.0000 | 0.0000 |
| *Le_*10007179 | GO:0005506//iron ion binding | 4.3496 | 5.3141 | 4.7899 | 2.3776 | 2.2100 | 3.2245 |
| *Le_*10007538 | GO:0005506//iron ion binding | 8.1010 | 8.6897 | 7.8551 | 3.7538 | 3.9018 | 4.6180 |
| *Le_*10007607 | GO:0008270//zinc ion binding | 5.8300 | 6.0198 | 5.6411 | 3.4721 | 3.3785 | 3.8751 |
| *Le_*10011313 | GO:0005506//iron ion binding | 0.0000 | 0.0931 | 0.2710 | 4.5317 | 3.9803 | 3.0761 |
| *Le_*10012618 | GO:0008270//zinc ion binding | 5.7409 | 5.4818 | 5.0688 | 2.9069 | 3.0727 | 3.4511 |
| *Le_*10003230 | [F]GO:0043565//sequence-specific DNA binding; GO:0003700//transcription factor activity, sequence-specific DNA binding; [P]GO:0006355//regulation of transcription, DNA-templated; | 2.8190 | 2.5000 | 2.2479 | 7.9482 | 7.7464 | 7.7759 |
| *Le_*10003441 | [F]GO:0043565//sequence-specific DNA binding; GO:0008270//zinc ion binding; GO:0003700//transcription factor activity, sequence-specific DNA binding; [P]GO:0006355//regulation of transcription, DNA-templated; | 1.5227 | 3.0012 | 2.9011 | 0.0000 | 0.0000 | 0.0000 |
| *Le_*10008419 | [F]GO:0043565//sequence-specific DNA binding; GO:0003700//transcription factor activity, sequence-specific DNA binding; [P]GO:0006355//regulation of transcription, DNA-templated; | 2.2006 | 2.3813 | 1.9782 | 4.5181 | 4.7429 | 4.5882 |
| *Le_*10008353 | [F]GO:0043565//sequence-specific DNA binding; GO:0008270//zinc ion binding; GO:0003700//transcription factor activity, sequence-specific DNA binding; [P]GO:0006355//regulation of transcription, DNA-templated; | 0.8426 | 0.8745 | 0.7570 | 1.9916 | 2.2152 | 2.0670 |
| *Le_*10003018 | [F]GO:0000981//RNA polymerase II transcription factor activity, sequence-specific DNA binding; GO:0008270//zinc ion binding; [P]GO:0006355//regulation of transcription, DNA-templated; [C]GO:0005634//nucleus; | 1.7239 | 2.5737 | 2.7754 | 0.0000 | 0.0000 | 0.0000 |
| *Le_*10000608 | [F]catalytic activity;binding; [P]metabolic process;metabolic process; cellular process; | 0.0000 | 1.6260 | 0.0000 | 0.7570 | 1.3084 | 1.5311 |
| *Le_*10002718 | [F]catalytic activity; binding; [C]organelle; macromolecular complex; organelle; organelle; macromolecular complex; cell; organelle; macromolecular complex; cell; cell part; organelle; macromolecular complex; cell; cell part; organelle part; | 1.5769 | 0.0000 | 1.2429 | 0.7712 | 0.7627 | 0.0473 |
| *Le_*10002733 | [F]structural molecule activity; binding; [C]organelle; macromolecular complex; organelle; organelle; macromolecular complex; cell; organelle; macromolecular complex; cell; cell part; organelle; macromolecular complex; cell; cell part; organelle part; [P]metabolic process; metabolic process; cellular process; | 4.3154 | 0.0000 | 4.1146 | 4.6464 | 3.5977 | 3.8676 |
| *Le_*10003351 | [F]binding; | 0.0000 | 2.2449 | 0.9107 | 2.0508 | 2.4436 | 2.2947 |
| *Le_*10004459 | [F]binding; | 1.0333 | 2.6841 | 1.0704 | 2.5801 | 2.8271 | 2.1055 |
| *Le_*10004619 | [F]catalytic activity; binding; | 0.0000 | 4.9114 | 3.0490 | 4.2658 | 4.4112 | 4.3198 |
| *Le_*10005960 | [F]catalytic activity; binding; [P]single-organism process; single-organism process; metabolic process; | 2.9585 | 4.8895 | 3.7122 | 0.0000 | 0.0000 | 0.0000 |
| *Le_*10009629 | [F]catalytic activity; binding; [P]single-organism process; metabolic process; single-organism process; metabolic process; cellular process; | 3.0203 | 6.3129 | 4.3142 | 4.3533 | 5.5665 | 1.0473 |
| *Le_*10010279 | [F]binding; | 2.5025 | 0.6354 | 1.3315 | 0.1506 | 0.5261 | 0.4294 |
| *Le_*10010560 | [F]binding; | 5.1445 | 0.0000 | 1.8600 | 0.0000 | 0.0000 | 3.9474 |
| *Le_*10011542 | [F]catalytic activity; binding; [P]single-organism process; single-organism process; metabolic process; | 1.0096 | 3.7141 | 2.2620 | 5.5012 | 5.8857 | 5.8750 |
| *Le_*10013693 | [F]catalytic activity; binding; [P]single-organism process; single-organism process; metabolic process; single-organism process; metabolic process; cellular process; | 0.0000 | 2.7775 | 2.1570 | 2.5977 | 2.8339 | 0.5753 |
| Membrane | | | | | | | |
| *Le_*10003014 | [C]GO:0016021//integral component of membrane; [P]GO:0006950//response to stress; | 0.8480 | 0.5850 | 0.3599 | 2.6151 | 2.2987 | 2.3074 |
| *Le_*10009935 | [F]GO:0004872//receptor activity; [C]GO:0016020//membrane; | 4.3246 | 4.0548 | 4.2785 | 7.1351 | 7.1272 | 6.9119 |
| *Le_*10004805 | [C]membrane; | 0.0000 | 4.6749 | 2.0096 | 3.1543 | 4.0218 | 2.7071 |
| Extracellular region |  |  |  |  |  |  |  |
| *Le_*10010320 | [P]GO:0009405//pathogenesis; [C]GO:0005576//extracellular region; | 7.5476 | 7.0234 | 7.6590 | 3.4423 | 3.8859 | 3.9721 |
| *Le_*10010322 | [P]GO:0009405//pathogenesis; [C]GO:0005576//extracellular region; | 0.0000 | 0.8319 | 0.0000 | 4.4603 | 4.7488 | 3.8768 |
| Localization | | | | | | | |
| *Le_*10001950 | [F]GO:0022857//transmembrane transporter activity; [P]GO:0055085//transmembrane transport; [C]GO:0016021//integral component of membrane; | 3.1032 | 3.1232 | 3.1662 | 0.0473 | 0.0426 | 0.5025 |
| *Le_*10002462 | [F]GO:0005215//transporter activity; [P]GO:0055085//transmembrane transport; [C]GO:0016021//integral component of membrane; | 4.1029 | 3.1331 | 4.1312 | 0.2790 | 0.1066 | 0.1848 |
| *Le_*10006364 | [F]GO:0022857//transmembrane transporter activity; [P]GO:0055085//transmembrane transport; [C]GO:0016021//integral component of membrane; | 4.9067 | 5.7391 | 5.3683 | 2.0886 | 1.6338 | 2.5882 |
| *Le_*10006934 | [P]GO:0055085//transmembrane transport; [C]GO:0016021//integral component of membrane; | 2.6773 | 2.6900 | 2.8453 | 5.2780 | 5.1221 | 5.6895 |
| *Le_*10007614 | [P]GO:0055085//transmembrane transport; | 1.2947 | 1.2610 | 1.7852 | 0.0886 | 0.2058 | 0.0566 |
| *Le_*10009468 | [F]GO:0022857//transmembrane transporter activity; [P]GO:0055085//transmembrane transport; [C]GO:0016021//integral component of membrane; | 3.3128 | 2.0179 | 3.0350 | 0.1066 | 0.0000 | 0.1419 |
| *Le_*10012169 | [P]GO:0055085//transmembrane transport; [C]GO:0016021//integral component of membrane; | 4.0403 | 3.5615 | 3.3089 | 6.7409 | 6.4826 | 6.7214 |
| *Le_*10013475 | [F]GO:0022857//transmembrane transporter activity; [P]GO:0055085//transmembrane transport; [C]GO:0016020//membrane;GO:0016021//integral component of membrane; | 6.6362 | 6.9865 | 6.9838 | 2.4708 | 2.5785 | 4.4256 |
| *Le_*10003609 | [F]GO:0022857//transmembrane transporter activity; [P]GO:0055085//transmembrane transport; [C]GO:0016021//integral component of membrane; | 1.6260 | 1.6900 | 1.0772 | 1.1419 | 0.4471 | 0.8074 |
| *Le_*10006404 | [F]GO:0022857//transmembrane transporter activity; [P]GO:0055085//transmembrane transport; [C]GO:0016020//membrane;GO:0016021//integral component of membrane; | 1.6945 | 1.6477 | 1.0215 | 1.6104 | 0.5327 | 1.1419 |
| *Le_*10009268 | [P]GO:0055085//transmembrane transport; [C]GO:0016021//integral component of membrane; | 1.6151 | 2.4276 | 1.0356 | 1.1995 | 0.7684 | 1.1506 |
| *Le_*10009463 | [P]GO:0055085//transmembrane transport; [C]GO:0016021//integral component of membrane; | 1.0589 | 1.8600 | 1.7880 | 0.9411 | 1.1656 | 0.4471 |
| *Le_*10014087 | [F]GO:0015095//magnesium ion transmembrane transporter activity; [C]GO:0016021//integral component of membrane; [P]GO:0015693//magnesium ion transport; | 0.2469 | 1.6292 | 0.0000 | 1.0024 | 1.6120 | 1.2750 |
| *Le_*10001594 | [F]transporter activity; [C]membrane; [P]localization; | 1.6229 | 3.6956 | 2.4418 | 4.5630 | 4.6114 | 4.8943 |
| *Le_*10001689 | [P]localization; [C]membrane;membrane;membrane part; | 2.3730 | 0.0000 | 0.9235 | 2.7648 | 0.7627 | 2.1088 |
| *Le_*10002245 | [P]localization; [C]membrane;membrane;membrane part; | 0.0000 | 4.1974 | 4.5940 | 0.6135 | 2.2957 | 0.1243 |
| *Le_*10008273 | [P]localization; [C]membrane;membrane;membrane part; | 0.0000 | 3.8898 | 3.2580 | 3.3780 | 3.8507 | 3.3368 |
| *Le_*10010327 | [F]transporter activity; [P]localization; [C]membrane; | 0.0000 | 3.9117 | 1.6736 | 2.7232 | 2.9075 | 1.1953 |
| Antioxidant activity | | | | | | | |
| *Le_*10000923 | [F]GO:0004601//peroxidase activity; | 1.0072 | 0.9461 | 0.9209 | 3.1795 | 3.2172 | 3.0993 |
| *Le_*10000926 | [F]GO:0004601//peroxidase activity; | 0.0000 | 0.0000 | 0.0520 | 2.5501 | 2.6485 | 2.2908 |
| *Le_*10004084 | [F]GO:0020037//heme binding; GO:0004601//peroxidase activity; [P]GO:0006979//response to oxidative stress;GO:0055114//oxidation-reduction process; | 0.0000 | 0.0000 | 0.0000 | 3.9669 | 3.6724 | 4.7097 |
| *Le_*10008662 |  |  |  |  |  |  |  |
| Catalytic activity | | | | | | | |
| *Le_*10011064 | [F]GO:0004222//metalloendopeptidase activity; | 0.1549 | 0.0000 | 0.0000 | 4.3067 | 4.4083 | 4.4575 |
| *Le_*10008442 | [F]GO:0016491//oxidoreductase activity; [P]GO:0008152//metabolic process; | 0.0000 | 0.0380 | 0.1806 | 2.9739 | 3.2126 | 3.0920 |
| *Le_*10008994 | [F]GO:0016491//oxidoreductase activity; [P]GO:0055114//oxidation-reduction process; | 5.3108 | 5.5472 | 5.6448 | 8.9356 | 8.9287 | 8.6236 |
| *Le_*10012258 | [F]GO:0016491//oxidoreductase activity; [P]GO:0055114//oxidation-reduction process; | 0.1419 | 0.0426 | 0.0426 | 2.8940 | 2.5882 | 4.1353 |
| *Le_*10007938 | [F]GO:0016627//oxidoreductase activity, acting on the CH-CH group of donors; [P]GO:0006629//lipid metabolic process; [C]GO:0016021//integral component of membrane;GO:0005737//cytoplasm; | 0.4612 | 0.2750 | 0.2987 | 2.7662 | 2.5025 | 2.7254 |
| *Le_*10002534 | [F]GO:0030599//pectinesterase activity; [P]GO:0042545//cell wall modification; [C]GO:0005618//cell wall; | 0.0000 | 0.0000 | 0.0000 | 2.1155 | 1.7181 | 1.5930 |
| *Le_*10008178 | [F]GO:0030599//pectinesterase activity; [P]GO:0042545//cell wall modification; [C]GO:0005618//cell wall; | 1.9733 | 2.2540 | 2.6096 | 0.3969 | 0.7796 | 0.3142 |
| *Le_*10010877 | [F]GO:0016491//oxidoreductase activity; [P]GO:0055114//oxidation-reduction process; | 1.6781 | 0.8823 | 1.5737 | 1.0473 | 0.4577 | 0.5753 |
| Signal transduction |  |  |  |  |  |  |  |
| *Le_*10009735 | [F]GO:0004435//phosphatidylinositol phospholipase C activity; [P]GO:0007165//signal transduction; GO:0035556//intracellular signal transduction;GO:0006629//lipid metabolic process; | 1.8745 | 4.9991 | 3.7276 | 1.8169 | 2.9702 | 0.1679 |
